# Markers of inflammation predict survival in newly diagnosed cirrhosis: a prospective registry study

**DOI:** 10.1038/s41598-023-47384-2

**Published:** 2023-11-16

**Authors:** Thit Mynster Kronborg, Henry Webel, Malene Barfod O’Connell, Karen Vagner Danielsen, Lise Hobolth, Søren Møller, Rasmus Tanderup Jensen, Flemming Bendtsen, Torben Hansen, Simon Rasmussen, Helene Bæk Juel, Nina Kimer

**Affiliations:** 1 Gastro Unit, Medical Division, Hvidovre University Hospital, Hvidovre, Denmark; 2 Novo Nordisk Foundation Centre for Protein Research, Copenhagen University, Copenhagen, Denmark; 3 Department of Clinical Physiology and Nuclear Medicine, Centre for Functional and Diagnostic Imaging and Research, Hvidovre Hospital, Hvidovre, Denmark; 4 Department of Clinical Medicine, Faculty of Health Sciences, University of Copenhagen, Copenhagen, Denmark; 5 Novo Nordisk Foundation Centre for Metabolic Research, Copenhagen University, Copenhagen, Denmark; 6 The Novo Nordisk Foundation Centre for Genomic Mechanisms of Disease, Broad Institute of MIT and Harvard, Cambridge, USA

**Keywords:** Physiology, Biomarkers, Gastroenterology, Medical research

## Abstract

The inflammatory activity in cirrhosis is often pronounced and related to episodes of decompensation. Systemic markers of inflammation may contain prognostic information, and we investigated their possible correlation with admissions and mortality among patients with newly diagnosed liver cirrhosis. We collected plasma samples from 149 patients with newly diagnosed (within the past 6 months) cirrhosis, and registered deaths and hospital admissions within 180 days. Ninety-two inflammatory markers were quantified and correlated with clinical variables, mortality, and admissions. Prediction models were calculated by logistic regression. We compared the disease courses of our cohort with a validation cohort of 86 patients with cirrhosis. Twenty of 92 markers of inflammation correlated significantly with mortality within 180 days (*q*-values of 0.00–0.044), whereas we found no significant correlations with liver-related admissions. The logistic regression models yielded AUROCs of 0.73 to 0.79 for mortality and 0.61 to 0.73 for liver-related admissions, based on a variety of modalities (clinical variables, inflammatory markers, clinical scores, or combinations thereof). The models performed moderately well in the validation cohort and were better able to predict mortality than liver-related admissions. In conclusion, markers of inflammation can be used to predict 180-day mortality in patients with newly diagnosed cirrhosis. Prediction models for newly diagnosed cirrhotic patients need further validation before implementation in clinical practice.

*Trial registration*: NCT04422223 (and NCT03443934 for the validation cohort), and Scientific Ethics Committee No.: H-19024348.

## Introduction

Cirrhosis of the liver is a condition with high mortality, and its incidence and prevalence are increasing worldwide^1–3^. Patients with cirrhosis can develop decompensation and are prone to infections, which causes frequent hospitalisations that place a considerable burden on health care systems^4^. To counter these challenges, we need more knowledge of risk profiles for patients at risk of admission and mortality.

The mechanisms driving acute decompensation have attracted increasing interest^5–8^. Inflammatory activity seems to be a major determinant in the mechanisms underlying the progression of cirrhosis, decompensation and related complications^7,9,10^. In alcohol-related liver disease, in particular, inflammation and oxidative stress seem to be prerequisites for the fibrogenesis that leads to cirrhosis. When cirrhosis progresses from the compensated to the decompensated stages, and to acute-on-chronic-liver failure (ACLF), systemic inflammation and immunodeficiency worsen, which is associated with a high risk of short-term mortality^11,12^. Indeed, it has been suggested that several markers of inflammation and cytokines are involved in driving acute decompensation and might be used to predict the risk of early death^10,13^. However, it is unknown whether markers can predict liver-related hospitalisations and mortality in patients with cirrhosis. Prospective studies with follow-up investigations of the relationship between markers of inflammation and prognoses of newly diagnosed cirrhosis are lacking, and the studies that have taken place are often from tertiary referral hospitals and based on selected patient groups^14,15^.

The aims of the present study were to assess whether systemic levels of inflammatory markers can predict liver-related admissions and short-term survival in newly diagnosed cirrhosis. We hypothesised, that markers of inflammation might predict survival and liver-related hospital admissions.

## Material and methods

### Participants

We recruited participants from a prospective disease registry study approved by the Scientific Ethics Committee of the Capital Region of Denmark (Reg. No: H-19024348) and registered with Clinicaltrials.gov (NCT04422223). Patients were recruited from a single university hospital with a catchment area of approximately 550,000 inhabitants in Copenhagen. All participants gave written informed consent for their participation, including informed consent to donate biological material for a research biobank. Thereby, the research was performed in accordance with all relevant regulations and in accordance with the Declaration of Helsinki.

Inclusion criteria were being older than 18 years; providing oral and written informed consent; and having a diagnosis of cirrhosis within the past 6 months based on unambiguous clinical, biochemical and imaging results, liver elastography, or histology verified by liver biopsy.

Exclusion criteria were a questionable diagnosis of liver cirrhosis or a diagnosis that was contradicted by histology; irreversible cognitive impairment; withdrawal of informed consent; or patients for whom the investigative program was delayed or not initiated within 6 months of their diagnosis.

### Data collection

Biochemistry, clinical data, comorbidities, and degree of decompensation at the time of diagnosis were registered at baseline. The study adhered to the Danish Data Protection Act (Reg. No: P-2019-545).

Mortality and admissions were registered prospectively using electronic patient records within 180 days of blood sampling for the biobank. Mortality and admissions were categorised as from “all causes” or “liver-related,” and an “unknown” cause of death indicated a patient had died outside the hospital and were not declared terminally ill beforehand. Liver-related events included liver-related infections (e.g., spontaneous bacterial peritonitis (SBP)) and complications, including ascites, hepatorenal syndrome (HRS), hepatic encephalopathy (HE), variceal bleeding, electrolyte disturbances and jaundice.

We used data from an earlier prospective study of patients with cirrhosis as a comparative validation cohort. Details about the study design and participant population have been published elsewhere^16^.

### Laboratory analyses of inflammation

The Target-96 Inflammation Panel (Olink Proteomics) semi-quantitatively measures 92 inflammatory markers (proteins) using proximity extension assay technology, which is based on qPCR extension of oligonucleotides attached to antibody pairs specific for the target protein^17^. Samples were split into two batches, and to minimise batch effects samples were randomised within a batch, and 16 bridging samples were included in each batch. Batches were bridged using the 16 bridging samples included in each batch. Samples flagged with quality control (QC) warnings in the standard Olink QC were reviewed manually before a decision was made on whether to retain or exclude them.

EDTA-plasma samples were thawed on ice and 40 μL of each was aliquoted into 96-well plates.

Raw data for normalised protein eXpression (NPX) values were processed in *R* using the OlinkAnalyze package^18^.

### Statistical analyses

**Figure 1:**
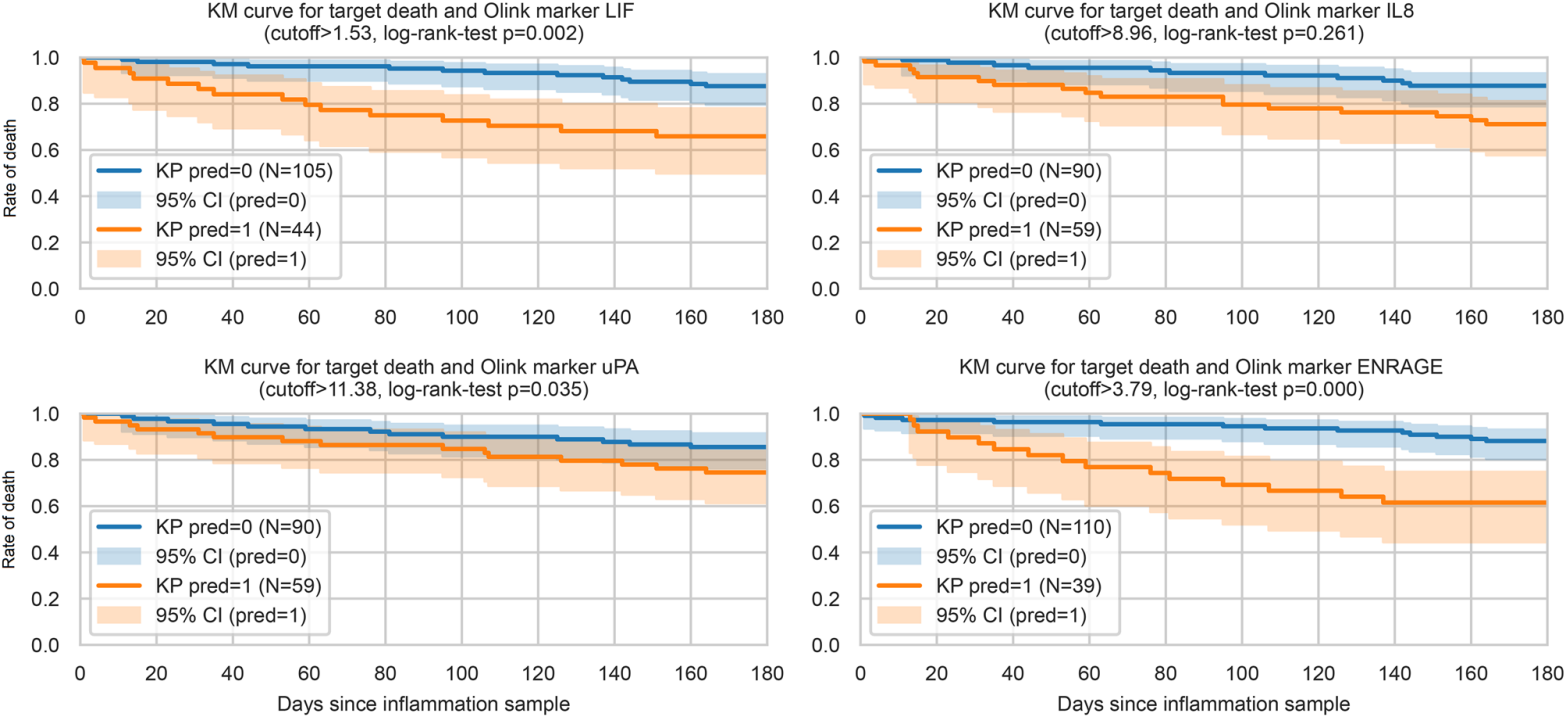
Kaplan–Meier curves for the four most significant inflammatory markers correlated with 180-day mortality. Concentrations of a marker higher than the cut-off are represented as orange curves, lower concentrations as blue curves. Pred = 1 indicates death within 180 days. Curves for each marker are compared by log rank tests.

We performed statistical analyses by multiple testing with corrected differential analyses. We looked at continuous clinical variables and clinical binary variables (uncontrolled) as well as inflammatory markers using a t-test or a binomial test^19,20^. The inflammatory markers univariate capability to separate death within 180 days was illustrated by survival curves, where two groups were separated based on a univariate logistic regression with an intercept (Fig. 1). Using the default cut-off for classification of *p* = 0.05 yields a cut-off corresponding to the negative fraction of the intercept and the coefficient of the marker. The log-rank-test was performed between the two predicted groups^20,21^. Further, the inflammatory (protein) markers were also compared using an analysis of covariance (ANCOVA) that controlled for covariates using a linear regression setup and the Benjamini–Hochberg procedure was used for multiple testing correction^20–22^. In the study group we controlled for sex, cancer, depression, psychiatric disorders, diabetes, heart disease, hypertension, and high cholesterol. We could not include cancer, depression, nor psychiatric disorders in the ANCOVA for our validation cohort due to an absence of information, and the inclusion criteria in the validation cohort excluded patients with any heart disease.

We used logistic regression for selected clinical features and protein markers of interest, with a selection of maximum relevance and minimum redundancy using the F-test-based implementation, specifically the F-test correlation quotient (FCQ), to choose a set of features for the logistic regression^21,23–25^. Results were calculated for the study group ten times using cross-validation, with five stratified splits for the target variable, i.e., 50 train-test split combinations. A final model was trained on the entire study group and compared to the validation cohort. We considered five feature sets: (1) three clinical scores (model for end-stage liver disease (MELD), MELD-sodium (MELD-Na) and Child–Pugh), with additional clinical markers (age, haemoglobin (Hgb), leucocytes, platelets, bilirubin, albumin, c-reactive protein (CRP), international normalised ratio (INR), alanine transaminase (ALT), creatinine, diabetes, arterial hypertension and hypercholesterolaemia), (2) 92 protein (inflammatory) markers, (3) inflammatory markers and clinical variables, (4) inflammatory markers and clinical scores and (5) the three clinical scores alone.

When suitable, adjusted* p*-values, so-called *q*-values were calculated to adjust for multiple testing using the Benjamini–Hochberg procedure. The adjustment reduces the number of false positive results.

Uniform manifold approximation and projection (UMAP) visualisation was based on the embeddings trained on 149 patients from the study group. The validation cohort were then mapped into the embedding space of the study group to highlight the relative locations^26^. Error analysis was based on the UMAP embedding, the prediction score and the classification error.

Performance was evaluated using several metrics. First, using the area under the receiver operating curves (AUROC’s), which shows the true positive rate relative to the false positive rate, which is one minus the true negative rate. Due to the imbalance between groups, we also considered the area under the curve of the precision-recall curve (AUPRC). Recall is also denoted as a true positive rate. A high PRAUC indicated that predictions were both precise and correct in most cases.

We are providing two open-source repositories for this study. First, one with higher-level scripts and workflows used for the analysis, github.com/RasmussenLab/cirrhosis_death. Second, we collected the core functionality for biomarker discovery, referred to as ‘not just another biomarker’ or ‘njab’, at github.com/RasmussenLab/njab. These results are reproducible and transferable to new datasets.

## Results

We recruited 151 patients for the study group at the Gastro Unit, Medical Division, Hvidovre Hospital, between 24 October 2019 and 31 March 2022. Follow-up ranged from 180 to 1028 days. Due to the length of time, we first analysed samples from 122 participants and then analysed samples from the remaining 29 participants. Statistics were performed on 149 patients as two were excluded due to metastatic cancer. Ninety-nine patients were male, and the median age was 62 years. Most patients had cirrhosis related to excessive alcohol consumption (128 = 85.9%), and the remaining aetiologies were metabolic associated steatohepatitis, viral hepatitis, autoimmune hepatitis, methotrexate-induced and haemochromatosis. The majority (109 = 73.2%) were diagnosed during hospitalisation, 37 (24.8%) of whom were incidentally diagnosed during admission for other diseases. Patients’ baseline characteristics are described in Table 1.

**Table 1:** Patient characteristics.

	Study group	Validation cohort	*p*-value, comparison of groups
	n = 149 (99 male)	n = 86 (51 male)	
Place of diagnosis			
Outpatient clinic	40 (26.8%)	NA	–
Admission at hospital	109 (73.2%)	NA	–
Age, years	62 [23;82]	61 [26;75]	0.329
Child–Pugh		0.003	
A	25 (16.8%)	31 (36.0%)	
B	68 (45.6%)	33 (38.4%)	
C	56 (37.6%)	22 (25.6%)	
Aetiology			
Alcohol	128 (85.9%)	70 (81.4%)	0.466
Non-alcohol	21 (14.1%)	16 (18.6%)	0.466
Decompensation at diagnosis	109 (73.2%)	NA(at sample time = 61 (70.9%))	(0.83)
Comorbidities			
Heart disease	27 (18.1%)	6 (7.3%)	0.040
Hypertension	25 (16.8%)	23 (28.4%)	0.057
Hypercholesterolaemia	13 (8.7%)	19 (22.6%)	0.006
Diabetes	27 (18.1%)	17 (20.2%)	0.824
Cancer	8 (5.4%)	NA	–
Psychiatric disease	13 (8.7%)	NA	–
Medication			
Statins	37 (24.8%)	19 (22.9%)	0.864
Non-selective beta-blockers	6 (4.0%)	21 (24.4%)	< 0.001
Biochemistry, median [range]			
Haemoglobin, mmol/L	7.1 [4.1;10.4]	7.40 [4.20, 10.30]	0.118
C-reactive protein, mg/L	14.0 [0.5;200]	5.80 [0.30, 110.00]	< 0.001
White blood cells, 10^9^/L	7.3 [1.7;71.3]	6.40 [2.20, 19.50]	0.012
Platelets, 10^9^/L	145 [33;461]	127.50 [29.00, 324.00]	0.102
Albumin, g/L	24 [10;42]	32.00 [15.00; 44.00]	< 0.001
Creatinine, µmol/L	64 [27;318]	71.00 [24.00, 358.00]	0.055
Bilirubin, µmol/L	29 [4;439]	18.00 [4.00, 362.00]	< 0.001
ALT, U/L	39 [7;648]	32.00 [1.30, 168.00]	0.030
INR	1.4 [0.9;2.7]	1.30 [0.90, 2.60]	0.002
MELD	14 [6;34]	10.79 [6.43, 39.00]	0.005
MELD-Na	17.5 [7.0;36.0]	13.19 [6.43, 40.00]	< 0.001

Groups are compared by a two-sided t-test for continuous variables and a binomial test for binary variables, reported in frequency and (percent).

Decompensation defined as: ascites, bleeding oesophageal varices, hepatorenal syndrome, hepatic encephalopathy or spontaneous bacterial peritonitis. *INR* International normalised ratio, *CRP* c-reactive protein, *MELD* model for end-stage liver disease, *MELD-Na* model for end-stage liver disease with sodium.

Comparative analyses were performed in a cohort of 86 participants with liver cirrhosis undergoing liver vein catheterisation. The validation cohort was recruited between April 2017 and 5 May 2021 (see Table 1). Albumin, prothrombin-proconvertin time (pp), sodium and potassium were higher in the validation cohort, whereas white blood cells, bilirubin, CRP, INR, ALT, MELD, MELD-Na, and Child–Pugh scores were higher in the study group. The number of admissions was not significantly different between the groups.

### Mortality

Within 180 days of follow-up, 28 (18.8%) participants died, 19 from liver-related causes (67.9%), hereof nine with ACLF.

Age was significantly higher in deceased patients within 180 days, as were MELD, MELD-Na, Child–Pugh, creatinine, and CRP. Ascites and jaundice correlated with death (see mean (SD) and *p*-values of the significant correlations in Table 2).

**Table 2:** Clinical and biochemical parameters significantly associated with 180-day mortality.

Variable	Deceased within 180 days (n = 28)		Survived within 180 days (n = 121)		*t*-test
	No. of patients	Mean (SD)	No. of patients	Mean (SD)	*p*-value
a)					
MELD-score	28	18.5 (6.1)	120	13.75 (5.56)	0.0006
Child–Pugh	28	9.9 (1.8)	121	8.57 (2.10)	0.0017
MELD-Na	28	21.0 (6.0)	121	17.09 (5.99)	0.003
Age	28	65.4 (9.1	121	60.6 (10.2)	0.018
Creatinine	28	99.4 (67.7)	120	70.93 (35.55)	0.039
CRP	28	39.5 (40.9)	118	22.81 (28.27)	0.048

Variable	Deceased within 180 days		Survived within 180 days		Binomial test
	No. of patients	Proportion	Proportion		*p*-value
b)					
Cancer	4	0.14	0.017		0.0011
Ascites	24	0.86	0.60		0.0036
Jaundice	9	0.32	0.15		0.0278

*CRP* c-reactive protein, *MELD* model of end-stage liver disease, *Na* sodium.

a) continuous variables, b) binary variables. Based on 149 patients’ continuous and binary variables. Two-sided *t*- and binomial tests.

### Liver-related admissions in the study group

Seven participants were excluded due to death during their admission at inclusion or when discharged with a terminal disease at baseline, leaving 142 participants for analysis.

Within 180 days of follow-up, 63 (44.4%) patients were admitted with a median time to first admission of 41 days (range [2;165]). Forty-five patients experienced liver-related admissions, with a median time to first admission of 40 days (range [2;161]). Infections caused 28 admissions (21.4% of all 180-day admissions), and 27 were liver-related infections.

### Clinical parameters and the risk of liver-related admissions

Significantly higher Child–Pugh and MELD scores were found in the 45 patients admitted with liver-related complications. Patients with subsequent liver-related death, ascites, who were unmarried, or had depression or decompensation at diagnosis were more prone to liver-related admission (Supplementary Table 1).

### Markers of inflammation analysis

Table 3 lists the inflammatory markers that were significantly correlated with 180-day mortality. In total, 20 markers differed significantly in patients who survived more than 180 days from those who did not. All but IL-12B were higher in the deceased. Correlations for all 92 markers are listed in Supplementary Table 2. The top four markers most strongly associated with mortality are shown in Fig. 1.

**Table 3:** Differential analysis of inflammatory markers significantly correlated with 180-day mortality.

Cell marker (NPX)	Deceased within 180 days, (n = 28)	Survived past 180 days, (n = 121)	Ancova	*q*-value
	Mean (SD)	Mean (SD)	Unadjusted *p*-value	
LIF	1.96 (1.27)	1.25 (0.49)	0	0
uPA	11.55 (0.55)	11.20 (0.52)	0	0.0009
IL-8	9.44 (1.53)	8.52 (1.39)	0.0001	0.0016
ENRAGE	4.46 (1.69)	3.33 (0.89)	0.0001	0.0018
TGFa	4.29 (0.72)	3.87 (0.49)	0.0001	0.0024
IL15-RA	3.01 (0.78)	2.61 (0.42)	0.0003	0.0049
IL12-B	6.29 (1.28)	6.95 (1.06)	0.0008	0.0103
MCP1	12.55 (0.68)	12.18 (0.56)	0.001	0.0114
CXCL6	10.36 (0.95)	9.90 (0.87)	0.0014	0.0136
HGF	11.21 (1.04)	10.72 (0.84)	0.0015	0.0136
CASP8	4.06 (0.47)	3.77 (0.51)	0.0016	0.0137
CX3CL1	6.10 (0.56)	5.76 (0.57)	0.0018	0.0139
IL10-RB	7.29 (0.27)	7.09 (0.31)	0.002	0.0144
CCL23	10.84 (0.66)	10.36 (0.61)	0.0025	0.0166
FGF19	10.20 (1.52)	9.42 (1.33)	0.0037	0.0226
IL-6	5.71 (1.41)	4.91 (1.19)	0.004	0.0232
TNFSF14	5.85 (0.84)	5.46 (0.79)	0.0046	0.024
ARTN	2.48 (0.89)	2.09 (0.59)	0.0047	0.024
MCP3	2.96 (0.90)	2.46 (0.64)	0.0064	0.031
CCL20	11.46 (1.61)	10.85 (1.33)	0.0096	0.044

*ARTN* artemin, *CASP8* caspase 8, *ENRAGE* extracellular newly identified receptor for advanced glycation end products binding protein, *HGF* hepatocyte growth factor, *IL* interleukin, *LIF* leukaemia inhibitory factor, *MCP1* C–C motif chemokine 2, *uPA* urokinase plasminogen activator, *MCP3* C–C motif chemokine 7, *CCL20* C–C motif chemokine 20.

Means of markers are expressed as NPX. N = 149 patients. Significant markers were found adjusting for sex, age, cancer, depression, psychiatric disease, diabetes, heart disease, hypertension and hypercholesterolaemia and their Ancova results are given in terms of unadjusted *p-*values.

No markers were significantly associated with liver-related admissions. The correlations are listed in Supplementary Table 3.

### Comparison with the validation cirrhosis cohort

In the validation cohort of 86 patients, the median age was 61 [26;75], 59.3% were male and the median MELD score was 10.0 [6;39] (see Table 1). Nine died within 180 days, and 32 were admitted a total of 76 times within 180 days. Twenty-six were admitted a total of 61 times for liver-related complaints. Four patients were excluded from the admission analysis due to death or because of missing data. The total number of deaths and admissions in both cohorts are listed in Supplementary Table 4a-b.

Two inflammatory markers, IL-15RA and IL-17C, were significantly associated with mortality in the validation cohort, with *q*-values of 0.024 and 0.030, respectively, only IL-15RA were also associated with mortality in the study group, see Supplementary Table 5.

No markers were significantly associated with admissions, see Supplementary Table 6.

### Prediction of mortality and liver-related admissions within 180 days in the study group

With data on inflammatory markers, clinical variables, standard biochemistry and clinical scores, several prediction models of mortality and admissions could be constructed. By cross-validation, we constructed five feature sets of variables to work as prediction models for 180-day mortality and liver-related admissions in the study group. The sets were: (1) clinical variables and clinical scores (MELD, MELD-Na and Child–Pugh), (2) inflammatory markers alone, (3) inflammatory markers and clinical variables, (4) inflammatory markers and clinical scores, and (5) clinical scores alone. The results of the prediction models are presented in Table 4, and correlations between included features are presented in Supplementary Fig. 1.

**Table 4:** Inflammatory and clinical variables as predictors of 180-day mortality and liver-related admissions.

Feature set	180-day mortality		Liver-related admission, 180 days	
	Markers/Variables	AUROC [CI95%]	Markers/Variables	AUROC [CI95%]
Inflammatory markers	ENRAGE, CD8A, IL-15RA, FGF19, LIF, ARTN, IL-12B	0.74 [0.46;1.02]	HGF, TRANCE, CCL4, IL-10RA, IL-15RA, SCF, TWEAK, FGF-19, CXCL6, IL-33, IL-10RB, IL-12B, CCL28, IL-2	0.72 [0.53;0.91]
Clinical variables	MELD, ascites, creatinine, age, CRP	0.79 [0.62;0.97]	Ascites, creatinine	0.64 [0.45;0.84]
Inflammatory markers + clinical variables and scores	ENRAGE, CD8A MELD-Na	0.75 [0.52;0.99]	TWEAK, CCL4, HGF, TRANCE, IL-33 Ascites	0.73 [0.57;0.88]
Inflammatory markers + scores	ENRAGE, CD8A MELD-Na	0.75 [0.52;0.99]	HGF, TRANCE, CCL4, IL-10RA, IL-15RA, SCF, TWEAK, FGF-19, CXCL6, IL-33, IL-10RB, IL-12B, CCL28, IL-2	0.72 [0.53;0.91]
Scores	MELD	0.73 [0.51;0.94]	Child–Pugh	0.61 [0.43;0.79]

Markers/variables: picked by models corresponding to the selected feature set.

*ARTN* artemin , *CCL-*CC motif chemokine ligand, *CD8A* T-cell surface glycoprotein CD8 alpha chain, *CRP* c-reactive protein, *CXCL6* C-X-C motif chemokine 6, *ENRAGE* Extracellular newly identified receptor for advanced glycation end products binding protein, *FGF19* Fibroblast growth factor 19*, HGF* hepatocyte growth factor, *IL* Interleukin, *LIF* leukaemia inhibitory factor, *MELD* Model for end-stage liver disease, *MELD-Na* Model for end-stage liver disease with sodium, *SCF* stem cell factor (a kit ligand), *TRANCE* Tumour necrosis factor ligand superfamily member 11, *TWEAK* Tumour necrosis factor ligand superfamily member 12.

The best models were a prediction model of the five clinical parameters MELD, ascites, creatinine, age and CRP (AUROC of 0.79 and 95% CI [0.62;0.97]) and a prediction model of two inflammatory markers and MELD-Na (AUROC of 0.75 and 95% CI [0.52;0.99]).

Among the clinical scores alone, MELD was the best predictor of mortality, with the AUROC being 0.73 (95% CI [0.51;0.94]).

Secondly, we investigated predictions of liver-related admissions within 180 days (see Table 4, Supplementary Fig. 2) using the same cross-validation approach we used for mortality. For the inflammatory markers alone, 14 markers were selected (HGF, TRANCE, CCL4, IL-10RA, IL-15RA, SCF, TWEAK, FGF-19, CXCL6, IL-33, IL-10RB, IL-12B, CCL28, IL-2), resulting in an AUROC of 0.72 with 95% CI [0.53;0.91]. Among clinical markers alone, two variables were selected (ascites and creatinine), giving an AUROC of 0.64 with 95% CI [0.45;0.84]. The combination of inflammatory markers and clinical variables favoured five inflammatory markers (TWEAK, CCL4, HGF, TRANCE and IL-33) and one clinical variable (ascites) with an AUROC of 0.73 and 95% CI [0.57;0.88]. Adding clinical scores to inflammatory markers did not change the results from using inflammatory markers alone. Predicting liver-related admissions from clinical scores alone was best achieved with Child–Pugh score alone, with an AUROC of 0.61 and 95% CI [0.43;0.79]. In general, the models predicting mortality were more reliable, with fewer selected features and higher AUROCs than models predicting liver-related admissions.

### Predictions in the validation cohort

In the study group, the clinical variables best able to predict death were MELD, ascites, creatinine, age, and CRP. Using the model trained on the study group with a cut-off of 0.5, we correctly predicted eight of nine deaths in the validation cohort, and incorrectly predicted that 13 of the 77 survivors would die. Inflammatory markers alone predicted six of nine deaths based on seven markers (ENRAGE, CD8A, IL-15RA, FGF19, LIF, ARTN and IL-12B), and incorrectly predicted ten survivors would die. The model was slightly improved when combining inflammatory markers and clinical variables and combining inflammatory markers with MELD-Na. The clinical scores favoured the MELD score alone, predicted eight deaths, and incorrectly predicted 12 patients would die, visualised in Supplementary Fig. 3.

Two clinical variables, ascites and creatinine, predicted 22 of 26 liver-related admissions, and incorrectly predicted that 31 admissions in the validation cohort would occur. Fourteen inflammatory markers (HGF, TRANCE, CCL4, IL-10RA, IL-15RA, SCF, TWEAK, FGF-19, CXCL6, IL-33, IL-10RB, IL-12B, CCL28, IL-2) predicted 17 of 26 liver-related admissions. Adding clinical scores to the inflammatory markers did not change the results. When we combined inflammatory markers and clinical variables, the model predicted 18 of 26 admissions. No model predicted all liver-related admissions correctly.

The full results of the cross-validation calculations in the study group and the validation cohort are presented in Supplementary Results 1a–e for 180-day mortality and Supplementary Results 2a–e for 180-day liver-related admissions.

### Visualizing predictions of samples

**Figure 2:**
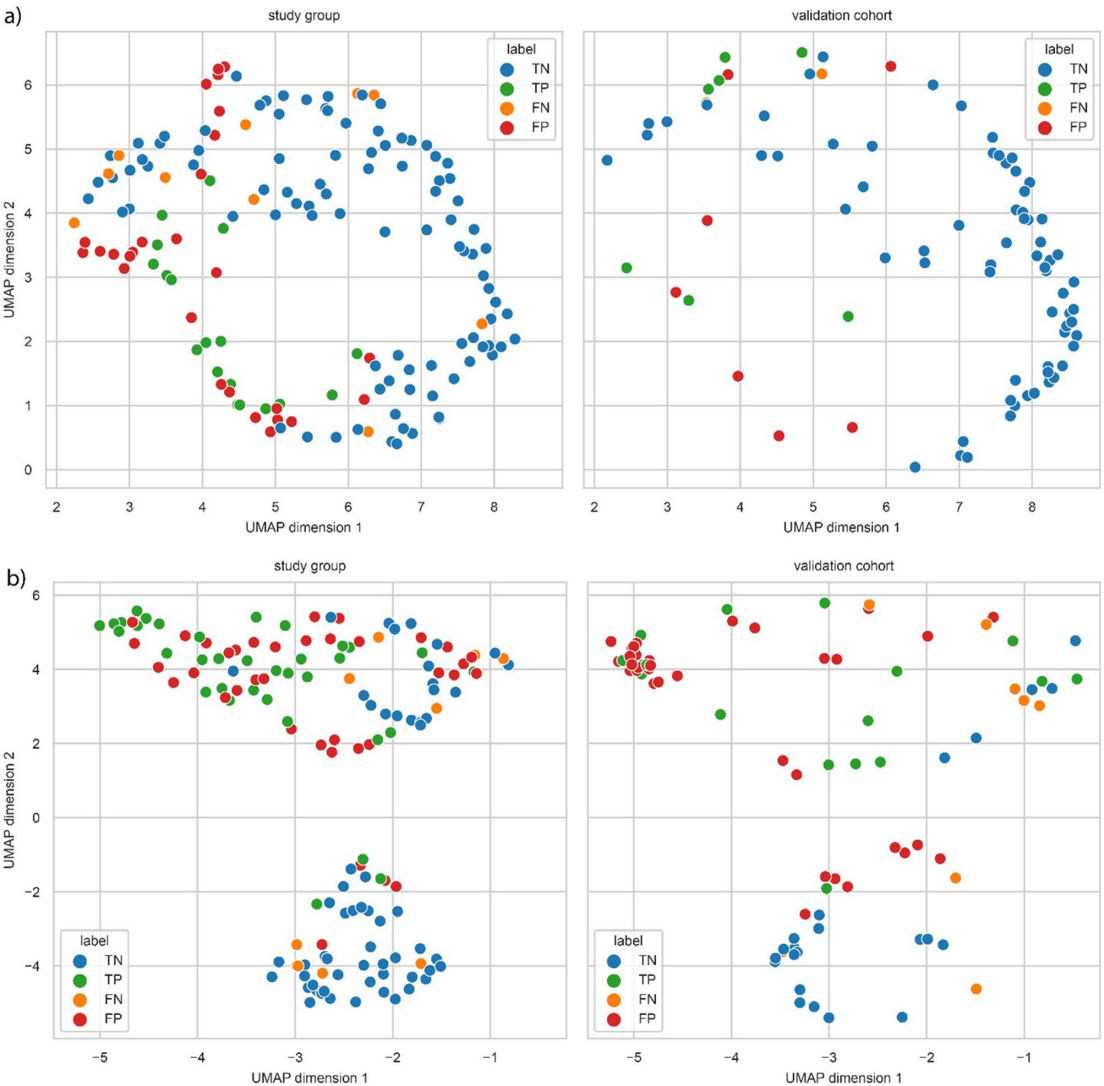
UMAP 2D-embedding of inflammatory markers among patients according to (**a**) 180-day mortality and (**b**) 180-day liver-related admissions, based on selected features for the model using clinical and inflammatory markers. Blue and red dots represent patients with no liver-related admissions, green and yellow dots represent patients who were admitted for liver-related reasons. FN, False negative; FP, False positive; TN, True negative; TP, True positive.

Further exploring the differences between our study group and the validation cohort, we investigated the numbers of true and false predictions of 180-day mortality and 180-day liver-related admissions using our final models, illustrated in Fig. 2a and b. Validation cohort samples are visualised in the two-dimensional mapping defined by the study group. False positive and false negative predictions were found in both the study group and the validation cohort, as indicated by closely clustered patients with different outcomes.

## Discussion

In this analysis of inflammatory markers in patients with newly diagnosed cirrhosis, we found 20 markers that correlated with mortality. The inflammatory markers differed from correlations in a validation cohort, and none correlated significantly with liver-related admissions within 180 days. Prediction models did not perform well in a comparative validation cohort of patients with cirrhosis.

The analyses provide exploratory insights into 92 different inflammatory markers. We expected markers associated with mortality and admissions to overlap due to our assumption of the correlation between increased risk of death and admissions in general^27^. Surprisingly, we did not find any markers of inflammation significantly associated with liver-related admissions in the study group, nor in the validation cohort. Furthermore, we found no generalisable marker pattern for predicting the endpoints.

Studies of unselected inflammatory markers with an explicit focus on newly diagnosed patients have to our knowledge previously not been performed. The focus of other studies has been on various stages of advanced liver disease^14,28,29^. Patients with compensated cirrhosis have an increased baseline inflammatory profile, and this increase becomes more pronounced in decompensated stages^14,29,30^. Among 29 cytokines investigated in the Canonic study, 15 cytokines, especially pro- and anti-inflammatory, were elevated in patients with decompensated cirrhosis and even higher in patients with ACLF^28^. Furthermore, different cytokine profiles were associated with different precipitating events for ACLF; bacterial infections caused elevated TNF-α, IL-6 and IL-1ra compared to alcohol, where IL-8 was especially elevated^28^.

Most of the markers we identified as related to the risk of 180-day mortality have formerly been associated with liver fibrosis and cirrhosis. The receptor of uPA (uPAR) is associated with fibrosis severity in liver diseases regardless of aetiology, and serum-uPAR concentrations are elevated in chronic liver diseases, with cirrhosis associated with the highest levels^31,32^. uPAR has also been used to predict mortality and the need for liver transplantation^32^. We have confirmed these findings for the activator, but not the receptor. Specific analyses of both receptors and activators are still needed.

LIF, a member of the IL-6 family, has previously been shown to correlate with the severity of liver steatosis^33^. IL-8 is a chemokine produced by a variety of cells, including liver cells. It is important for immune cell chemotaxis and can be used to predict mortality in ACLF^34^, why it might be related to the development of liver fibrosis and contribute to hepatic inflammation in chronic liver diseases^32,35^. Concurring with the earlier evidence, we found increased levels of LIF and IL-8 in our deceased participants.

TNF-cytokines, IL-1β, IL-6, IFN-γ, IL-17, MCP1, MIP1b and TNF-soluble receptors have been shown to be elevated in compensated and decompensated cirrhosis^11^. IL-17 was elevated in patients with alcoholic hepatitis, enhanced liver injury and inflammation^36^, which increases the risk of mortality. Trebicka et al. found higher levels of IL-6, IL-8, MCP1, IL-17A and IL-10 in patients with ACLF than in patients without ACLF, and an associated increased risk of death within 90 days^30^. Independent risk markers of death were IL-8 and human non-mercaptalbumin 2 (HNA2). We found higher levels of TNFSF14, IL-6, MCP1 and MCP3 in participants who died than in survivors, which could explain the differences between markers as prognostic indicators. In addition, we found elevated IL-17C levels in patients who died within 180 days in the validation cohort. IL-8 levels were associated with 180-day mortality, but our analysis did not include HNA2; hence, IL-8 and HNA2, in combination, should be investigated in future studies.

ENRAGE has been shown to be higher in patients with autoimmune hepatitis (AIH) than in healthy individuals, with the highest values seen in the presence of cirrhosis^37^, but it has been little studied in other causes of cirrhosis. Among others, IL-15, CX3CL1 and IL-6 were elevated in patients with minimal HE^38^, a cognitive impairment we did not assess, but which is likely present in our groups, and the markers could be correlated with both HE and death.

IL-10 is generally considered an anti-inflammatory cytokine^13^; however, IL-10 was higher in our deceased participants than in survivors, which might be evidence of a compensatory up-regulation. Kronsten et al.^29^ found lower levels of IL-10 in blood from patients with cirrhosis compared to healthy individuals; however, IL-10 increased, along with IL-1β, IL-6, TNF-α, IL-8 and IFNs, when stimulated by heat-killed Escherichia choli and lipopolysaccharides, indicating increases of IL-10 when inflammatory activity is high.

IL-6 has been shown to predict the first decompensation episodes and 1-year mortality in decompensated liver disease, and is associated with liver fibrogenesis in stable cirrhosis and portal hypertension^14,39^. Our findings support IL-6 as a marker of disease severity in cirrhosis, as it was significantly elevated in our deceased participants.

Prediction models achieved AUROCs ranging from 0.73 to 0.79 for mortality and from 0.61 to 0.73 for liver-related admissions, depending on the feature sets (clinical vs. inflammatory markers, or combinations thereof) in a cross-validation procedure on the study group. Hence, our models would score a patient who dies higher in at least 73% of the cases than a patient who survives when predicting 180-day mortality. As all the AUROCs were within a narrow range, we cannot conclude that one data modality (inflammatory markers or clinical data), or one specific combination, is superior for predicting mortality or admissions.

Interestingly, our AUROC’s are similar to the 90-day prediction by MELD in a cohort of unselected patients with cirrhosis^40^. Furthermore, in a large-scale review of predictions of 180-day mortality in cirrhosis by Child–Pugh and MELD, AUC-values ranged between 0.63 and 0.91 and 0.73–0.98, respectively^41^. Future studies could help pinpoint clinically useful scores, and these preliminary models might be optimised with differentiation according to disease severity in larger cohorts.

The UMAPs (Fig. 2a and b) show varying degrees of clustering in the predictions for mortality and admissions in both the study group and the validation cohort. Although most participants had alcohol as the aetiology of their cirrhosis, other causes were also present, which might have contributed to these broad variations in inflammation. In addition, a large proportion had ongoing episodes of decompensation when the blood sample was drawn, which could have impacted inflammatory activity. Further studies should look at how the various causes and stages of cirrhosis affect the concentration of individual markers of inflammation.

The MELD score performed better in the validation cohort than in the newly diagnosed cirrhosis study group. The differences in the groups, time of diagnosis, and the presence of complications and comorbidities, can likely help explain this discrepancy.

Finally, the error analysis supports more accurate predictions of death in the validation cohort, see UMAP in Fig. 2a. Patients have visually divided more accurately according to the outcome than our primary study group. However, the predictions for admissions proved less reliable (Fig. 2b). This is valuable knowledge, as treating patients with newly diagnosed versus established cirrhosis are two different disciplines.

Our study has several limitations, as a disease registry study with baseline sampling and no intervention. Prospective follow-up was collected from patient journals and intervention effects could not be assessed, such as antibiotic treatment of infections, albumin and alcohol abstinence. Ongoing infection at inclusion was not a reason for exclusion, which undoubtedly affected the levels of circulating inflammatory markers. Furthermore, we do not have follow-up data about the markers. Exclusion criteria for the validation cohort excluded heart- and kidney disease as well as clinical signs of cancer. The difference in life expectancy among patients with cirrhosis might have influenced our comparisons, since short-term mortality was higher in the study group. In addition, we saw significant differences in Child–Pugh scores and the use of beta-blockers between the groups.

Analyses of inflammatory markers were performed in two batches, so we cannot exclude minor batch effects, despite controlling for these using identical bridge samples in both. The number of participants in our study is too small to establish prediction models appropriate for clinical application. Nonetheless, all data were collected prospectively from a single centre that is representative of Denmark’s newly diagnosed population with cirrhosis, reflecting the real-world epidemiology of cirrhosis.

In conclusion, circulating markers of inflammation correlate with 180-day mortality in patients with cirrhosis. The performance of models in the validation cohort was moderate, with predictions of mortality superior to predictions of hospital admission. However, these models need further validation in larger cohorts before they reach clinical application.

### Supplementary Information

Supplementary Information 1.

Supplementary Information 2.

Supplementary Information 3.

Supplementary Information 4.

Supplementary Information 5.

Supplementary Information 6.

Supplementary Information 7.

Supplementary Information 8.

Supplementary Information 9.

Supplementary Information 10.

Supplementary Information 11.

## Data Availability

The complete statistical results are attached as Supplementary Results Files, Supplementary Material and are available, along with complete statistical codes, at github.com/RasmussenLab/cirrhosis_death and github.com/RasmussenLab/njab. In addition, the study protocol, standard operating procedures and patient information are available upon request to the authors. Any data provided must not be processed for purposes other than statistical and scientific studies.

## Abbreviations

ACLF: Acute-on-chronic liver failure
AIH: Autoimmune hepatitis
ALT: Alanine transaminase
ARTN: Artemin
AUROC: Area under the receiver operating curve
AXIN1: Axin-1
CASP8: Caspase 8
CCL-: CC motif chemokine ligand
CD8A: T-cell surface glycoprotein CD8 alpha chain
CRP: C-reactive protein
CX3CL1: Fractalkine, inflammatory marker
CXCL6: C-X-C motif chemokine 6
ENRAGE: Extracellular newly identified receptor for advanced glycation end products-binding protein
FGF19: Fibroblast growth factor 19
HCC: Hepatocellular carcinoma
HE: Hepatic encephalopathy
HGF: Hepatocyte growth factor
HNA2: Human non-mercaptalbumin 2
HRS: Hepatorenal syndrome
IL-6: Interleukin 6
IL-10RB: Interleukine 10 receptor subunit β
INR: International normalised ratio
LIF: Leukemia inhibitory factor
LoD: Limit of detection
MCP1: C–C motif chemokine 2
MCP3: C–C motif chemokine 3
MELD: Model for end-stage liver disease
MELD-Na: Model for end-stage liver disease with sodium
MIP1b: Macrophage inflammatory protein-1b
NAFLD: Non-alcoholic fatty liver disease
NASH: Non-alcoholic steatohepatitis
NPX: Normalised protein eXpression
pp: Prothrombin-proconvertin time
ROC: Receiver operating curve
SBP: Spontaneous bacterial peritonitis
SCF: Stem cell factor (a kit ligand)
TGF: Transforming growth factor
TNFSF14: Tumour necrosis factor ligand superfamily member 14
TRANCE: Tumour necrosis factor ligand superfamily member 11
TWEAK: Tumour necrosis factor ligand superfamily member 12
UMAP: Uniform manifold approximation and projection
uPA: Urokinase plasminogen activator
