# Supplementary material for: Markers of inflammation predict survival in newly diagnosed cirrhosis: a prospective registry study
Source: Sci Rep. 2023 Nov 16;13:20039. doi: 10.1038/s41598-023-47384-2 (PMC10654496; doi:10.1038/s41598-023-47384-2)

**Supplementary Material**

**Markers of inflammation predict survival in newly diagnosed cirrhosis: a prospective registry study**

Thit Mynster Kronborg*^1^, Henry Webel*^2^, Malene Barfod O’Connell^1^, Karen Vagner Danielsen^1^, Lise Hobolth^1^, Søren Møller^3,4^, Rasmus Tanderup Jensen^5^, Flemming Bendtsen^1,4^, Torben Hansen^5^, Simon Rasmussen^2,6^, Helene Bæk Juel^5^, Nina Kimer^1^

*Indicates shared first authorship

1. Hvidovre University Hospital, Gastro Unit, Medical Division

2. Novo Nordisk Foundation Centre for Protein Research, Copenhagen University

3. Centre for Functional and Diagnostic Imaging and Research, Department of Clinical Physiology and Nuclear Medicine, Hvidovre Hospital, Hvidovre, Denmark

4. Department of Clinical Medicine, Faculty of Health Sciences, University of Copenhagen

5. Novo Nordisk Foundation Centre for Metabolic Research, Copenhagen University

6. The Novo Nordisk Foundation Centre for Genomic Mechanisms of Disease, Broad Institute of MIT and Harvard, Cambridge, USA

Corresponding author: Thit Mynster Kronborg, thit.mynster.kronborg@regionh.dk

**Supplementary Tables**

**Supplementary Table 1) Clinical variables significantly associated with liver-related admissions within 180 days** of n=143, **a) continuous variables, b) binary variables;** Two-sided t- and binomial tests. *MELD: model of end-stage liver disease*

| **a)** | **Liver-rel. admission** | | | **No liver-rel. admission** | | **t-test** |
| --- | --- | --- | --- | --- | --- | --- |
| **Variable** | **No. Patients** | | **Mean (SD)** | **No. Patients** | **Mean (SD)** | **p-value** |
| **ChildPugh** |  | 45 | 9.36 (1.87) | 97 | 8.47 (2.15) | 0.015 |
| **MELD-score** |  | 45 | 15.96 (5.98) | 96 | 13.76 (5.82) | 0.044 |

| **b)** | **Liver-rel. admission (n=45)** | | **No liver-rel. admission (n=98)** | **Binomial test** |
| --- | --- | --- | --- | --- |
| **Variable** | **No. of patients** | **Proportion** | **Proportion** | **p-value** |
| **LiverRelated death** | 17 | 0.3778 | 0.0722 | 0 |
| **Ascites** | 36 | 0.8 | 0.546 | 0.0005 |
| **Unmarried** | 13 | 0.2889 | 0.1443 | 0.0102 |
| **DecomensatedAtDiagnosis** | 38 | 0.8444 | 0.598 | 0.0073 |
| **Depression** | 3 | 0.0667 | 0.0103 | 0.0113 |

**Supplementary Table 2) Markers of inflammation correlated to all-cause death within 180 days. ANCOVA analysis**. 92 markers. Controlled for sex, age, cancer, depression, psychiatric disorder, diabetes, heart disease, hypertension and hypercholesterolaemia.

| **event** | **Deceased within 180 days** | | | **Survival past 180 days** | | | **t-test** | | **Ancova** | | | |
| --- | --- | --- | --- | --- | --- | --- | --- | --- | --- | --- | --- | --- |
| **stats** | **count** | **mean** | **std** | **count** | **mean** | **std** | **p-val** | **cohen-d** | **p-unc** | **np2** | **-Log10  pvalue** | **qvalue** |
| **variable** |  |  |  |  |  |  |  |  |  |  |  |  |
| **LIF** | 28 | 1,962 | 1,2681 | 121 | 1,2506 | 0,4865 | 0,0067 | 1,0179 | 0 | 0,177 | 6,6427 | 0 |
| **uPA** | 28 | 11,5524 | 0,5484 | 121 | 11,1994 | 0,5158 | 0,0036 | 0,6765 | 0 | 0,1238 | 4,6939 | 0,0009 |
| **IL8** | 28 | 9,4401 | 1,5261 | 121 | 8,524 | 1,3947 | 0,006 | 0,6453 | 0,0001 | 0,1122 | 4,281 | 0,0016 |
| **ENRAGE** | 28 | 4,4569 | 1,686 | 121 | 3,3277 | 0,8852 | 0,0017 | 1,0476 | 0,0001 | 0,1073 | 4,1092 | 0,0018 |
| **TGFa** | 28 | 4,2928 | 0,721 | 121 | 3,872 | 0,4943 | 0,0061 | 0,7748 | 0,0001 | 0,1007 | 3,8759 | 0,0024 |
| **IL15RA** | 28 | 3,0092 | 0,7757 | 121 | 2,6089 | 0,4194 | 0,0128 | 0,7943 | 0,0003 | 0,09 | 3,4983 | 0,0049 |
| **IL12B** | 28 | 6,2856 | 1,2828 | 121 | 6,9459 | 1,0608 | 0,0159 | 0,5977 | 0,0008 | 0,0788 | 3,1066 | 0,0103 |
| **MCP1** | 28 | 12,5503 | 0,6778 | 121 | 12,18 | 0,5552 | 0,0108 | 0,6389 | 0,001 | 0,0759 | 3,0052 | 0,0114 |
| **CXCL6** | 28 | 10,3605 | 0,9469 | 121 | 9,902 | 0,8689 | 0,0244 | 0,5188 | 0,0014 | 0,0713 | 2,8474 | 0,0136 |
| **HGF** | 28 | 11,2123 | 1,0389 | 121 | 10,7228 | 0,8421 | 0,026 | 0,5553 | 0,0015 | 0,0709 | 2,8305 | 0,0136 |
| **CASP8** | 28 | 4,062 | 0,4743 | 121 | 3,7715 | 0,5071 | 0,0062 | 0,5796 | 0,0016 | 0,0696 | 2,7869 | 0,0137 |
| **CX3CL1** | 28 | 6,1009 | 0,5643 | 121 | 5,7624 | 0,5743 | 0,0068 | 0,5912 | 0,0018 | 0,0683 | 2,7402 | 0,0139 |
| **IL10RB** | 28 | 7,2886 | 0,2747 | 121 | 7,0917 | 0,3143 | 0,0018 | 0,6404 | 0,002 | 0,0669 | 2,6907 | 0,0144 |
| **CCL23** | 28 | 10,8438 | 0,6581 | 121 | 10,3551 | 0,6082 | 0,0009 | 0,7912 | 0,0025 | 0,0642 | 2,5966 | 0,0166 |
| **FGF19** | 28 | 10,2032 | 1,5184 | 121 | 9,4156 | 1,3323 | 0,0158 | 0,5756 | 0,0037 | 0,0595 | 2,4331 | 0,0226 |
| **IL6** | 28 | 5,7142 | 1,4123 | 121 | 4,91 | 1,1918 | 0,0083 | 0,6511 | 0,004 | 0,0583 | 2,3938 | 0,0232 |
| **TNFSF14** | 28 | 5,8525 | 0,838 | 121 | 5,4558 | 0,7934 | 0,0282 | 0,4948 | 0,0046 | 0,0566 | 2,333 | 0,024 |
| **ARTN** | 28 | 2,4825 | 0,8885 | 121 | 2,0926 | 0,5878 | 0,034 | 0,5967 | 0,0047 | 0,0565 | 2,329 | 0,024 |
| **MCP3** | 28 | 2,9623 | 0,9049 | 121 | 2,458 | 0,6372 | 0,0086 | 0,7266 | 0,0064 | 0,0526 | 2,1919 | 0,0311 |
| **CCL20** | 28 | 11,4626 | 1,6072 | 121 | 10,8473 | 1,3257 | 0,0678 | 0,4454 | 0,0096 | 0,0476 | 2,0182 | 0,0441 |
| **OSM** | 28 | 5,037 | 1,055 | 121 | 4,6807 | 0,9615 | 0,11 | 0,3638 | 0,0128 | 0,0441 | 1,8924 | 0,0561 |
| **CSF1** | 28 | 11,0024 | 0,2243 | 121 | 10,899 | 0,2128 | 0,0323 | 0,4811 | 0,0144 | 0,0426 | 1,8424 | 0,0601 |
| **IL18** | 28 | 10,3075 | 0,6928 | 121 | 10,0213 | 0,7729 | 0,0605 | 0,3773 | 0,0202 | 0,0385 | 1,6939 | 0,0809 |
| **CDCP1** | 28 | 5,3532 | 0,8766 | 121 | 5,0904 | 0,8584 | 0,1591 | 0,3049 | 0,0261 | 0,0353 | 1,583 | 0,0986 |
| **IL17C** | 28 | 4,6868 | 1,3945 | 121 | 4,1181 | 1,0167 | 0,0496 | 0,519 | 0,0268 | 0,035 | 1,572 | 0,0986 |
| **VEGFA** | 28 | 11,7439 | 0,6048 | 121 | 11,5071 | 0,4747 | 0,0606 | 0,4727 | 0,0287 | 0,0342 | 1,5415 | 0,1017 |
| **FGF23** | 28 | 5,2986 | 2,1206 | 121 | 4,4636 | 1,5833 | 0,0581 | 0,4927 | 0,0365 | 0,0313 | 1,4381 | 0,1243 |
| **NT3** | 28 | 2,744 | 0,6428 | 121 | 2,9936 | 0,5599 | 0,0659 | 0,4333 | 0,0378 | 0,0309 | 1,4221 | 0,1243 |
| **CD5** | 28 | 6,5747 | 0,7661 | 121 | 6,3097 | 0,5109 | 0,0907 | 0,4679 | 0,0425 | 0,0295 | 1,3716 | 0,1348 |
| **TNFB** | 28 | 5,0686 | 0,6947 | 121 | 5,323 | 0,6413 | 0,0846 | 0,3904 | 0,0505 | 0,0274 | 1,2968 | 0,1548 |
| **IL1_a** | 28 | -0,5642 | 0,6406 | 121 | -0,7422 | 0,3956 | 0,1684 | 0,3949 | 0,0678 | 0,024 | 1,1689 | 0,2012 |
| **OPG** | 28 | 11,4539 | 0,4225 | 121 | 11,2672 | 0,4659 | 0,0448 | 0,4075 | 0,071 | 0,0234 | 1,1485 | 0,2041 |
| **IL18R1** | 28 | 9,8433 | 0,7218 | 121 | 9,6757 | 0,6234 | 0,2639 | 0,2608 | 0,0732 | 0,0231 | 1,1355 | 0,2041 |
| **LAP_TGFb1** | 28 | 7,4416 | 0,4644 | 121 | 7,3279 | 0,4351 | 0,2448 | 0,258 | 0,0803 | 0,022 | 1,0956 | 0,2127 |
| **MMP1** | 28 | 14,5058 | 0,9342 | 121 | 14,1925 | 1,1311 | 0,1318 | 0,2855 | 0,0809 | 0,0219 | 1,092 | 0,2127 |
| **CXCL1** | 28 | 10,0302 | 1,2037 | 121 | 9,6962 | 1,0842 | 0,186 | 0,3016 | 0,0899 | 0,0207 | 1,0462 | 0,2235 |
| **Flt3L** | 28 | 9,3998 | 0,6562 | 121 | 9,6304 | 0,5915 | 0,0961 | 0,382 | 0,0934 | 0,0203 | 1,0295 | 0,2235 |
| **CD40** | 28 | 12,5945 | 0,6114 | 121 | 12,3697 | 0,499 | 0,0786 | 0,4311 | 0,0939 | 0,0202 | 1,0274 | 0,2235 |
| **4EBP1** | 28 | 9,8006 | 0,9633 | 121 | 9,4943 | 0,948 | 0,1362 | 0,3221 | 0,0948 | 0,0201 | 1,0234 | 0,2235 |
| **IFNg** | 28 | 7,7385 | 1,225 | 121 | 8,2552 | 1,5205 | 0,0613 | 0,3513 | 0,1021 | 0,0192 | 0,991 | 0,2348 |
| **AXIN1** | 28 | 5,058 | 1,1356 | 121 | 5,4265 | 1,1894 | 0,1327 | 0,3124 | 0,1126 | 0,0181 | 0,9484 | 0,2518 |
| **MMP10** | 28 | 10,289 | 0,8441 | 121 | 10,0566 | 0,7529 | 0,1887 | 0,3016 | 0,1149 | 0,0179 | 0,9395 | 0,2518 |
| **CXCL9** | 28 | 7,9039 | 0,7536 | 120 | 7,976 | 1,0654 | 0,6775 | 0,071 | 0,1212 | 0,0174 | 0,9166 | 0,2579 |
| **TRAIL** | 28 | 7,2235 | 0,4 | 121 | 7,3398 | 0,4103 | 0,1751 | 0,2847 | 0,1234 | 0,0171 | 0,9088 | 0,2579 |
| **IL10RA** | 28 | 2,1599 | 0,6572 | 121 | 1,974 | 0,5492 | 0,1734 | 0,3258 | 0,127 | 0,0168 | 0,8961 | 0,259 |
| **DNER** | 28 | 8,8239 | 0,4524 | 121 | 8,7502 | 0,3811 | 0,4294 | 0,1866 | 0,1295 | 0,0166 | 0,8877 | 0,259 |
| **SCF** | 28 | 7,3767 | 0,5334 | 121 | 7,5444 | 0,9719 | 0,215 | 0,1848 | 0,1558 | 0,0145 | 0,8074 | 0,305 |
| **CCL3** | 28 | 7,1892 | 0,6948 | 121 | 6,9003 | 0,7795 | 0,0592 | 0,3778 | 0,1602 | 0,0142 | 0,7954 | 0,307 |
| **PDL1** | 28 | 6,9227 | 0,4591 | 121 | 6,765 | 0,4722 | 0,111 | 0,3356 | 0,1699 | 0,0136 | 0,7698 | 0,319 |
| **TRANCE** | 28 | 4,8489 | 0,8088 | 121 | 4,7079 | 0,7509 | 0,4049 | 0,185 | 0,19 | 0,0124 | 0,7212 | 0,3496 |
| **BetaNGF** | 28 | 0,3817 | 0,1952 | 121 | 0,3263 | 0,2259 | 0,1959 | 0,2513 | 0,1981 | 0,012 | 0,7032 | 0,3547 |
| **CD8A** | 28 | 11,2742 | 0,8204 | 121 | 11,0235 | 0,8131 | 0,1521 | 0,3078 | 0,2023 | 0,0118 | 0,6941 | 0,3547 |
| **IL22_RA1** | 28 | 3,0284 | 0,4923 | 121 | 2,9591 | 0,5407 | 0,5135 | 0,1303 | 0,2044 | 0,0116 | 0,6896 | 0,3547 |
| **IL2RB** | 28 | 1,8484 | 1,0041 | 121 | 1,6388 | 0,5328 | 0,293 | 0,3245 | 0,2177 | 0,011 | 0,6622 | 0,3708 |
| **CCL25** | 28 | 7,415 | 0,7191 | 121 | 7,2688 | 0,5764 | 0,3223 | 0,2416 | 0,2317 | 0,0103 | 0,635 | 0,3852 |
| **ADA** | 28 | 6,3253 | 0,6296 | 121 | 6,1807 | 0,56 | 0,2708 | 0,2522 | 0,2345 | 0,0102 | 0,6299 | 0,3852 |
| **IL33** | 28 | 1,3822 | 0,3478 | 121 | 1,3047 | 0,2734 | 0,2775 | 0,2687 | 0,24 | 0,01 | 0,6199 | 0,3873 |
| **TWEAK** | 28 | 8,7466 | 0,6076 | 121 | 8,6187 | 0,5072 | 0,3083 | 0,2426 | 0,2576 | 0,0093 | 0,5891 | 0,4085 |
| **CD6** | 28 | 7,2658 | 0,9656 | 121 | 7,1272 | 0,7185 | 0,4793 | 0,1801 | 0,2684 | 0,0089 | 0,5712 | 0,4186 |
| **IL24** | 28 | 2,4247 | 0,6597 | 117 | 2,2519 | 0,6967 | 0,2251 | 0,2504 | 0,2746 | 0,0089 | 0,5613 | 0,421 |
| **NRTN** | 28 | 1,4539 | 0,594 | 121 | 1,4446 | 0,5843 | 0,9407 | 0,0159 | 0,3261 | 0,007 | 0,4866 | 0,4919 |
| **GDNF** | 28 | 3,8072 | 0,739 | 121 | 3,7559 | 0,5871 | 0,7337 | 0,083 | 0,3395 | 0,0066 | 0,4692 | 0,5037 |
| **CXCL10** | 28 | 11,001 | 0,7487 | 121 | 11,1134 | 1,0339 | 0,5112 | 0,1138 | 0,3501 | 0,0063 | 0,4559 | 0,5112 |
| **FGF21** | 28 | 8,2644 | 2,7635 | 121 | 8,0731 | 2,4056 | 0,7373 | 0,0773 | 0,3758 | 0,0057 | 0,425 | 0,5402 |
| **LIFR** | 28 | 5,1424 | 0,3789 | 121 | 5,0801 | 0,3562 | 0,4333 | 0,1726 | 0,3976 | 0,0052 | 0,4006 | 0,5627 |
| **IL4** | 28 | 1,2806 | 0,8394 | 121 | 1,192 | 0,9022 | 0,6225 | 0,0994 | 0,486 | 0,0035 | 0,3133 | 0,673 |
| **CCL28** | 28 | 4,3315 | 0,5996 | 121 | 4,243 | 0,5914 | 0,4843 | 0,1493 | 0,4917 | 0,0034 | 0,3083 | 0,673 |
| **CCL4** | 28 | 6,711 | 0,6391 | 121 | 6,592 | 0,9119 | 0,4204 | 0,137 | 0,4983 | 0,0033 | 0,3025 | 0,673 |
| **IL17A** | 27 | 3,3525 | 1,0837 | 121 | 3,1087 | 0,9669 | 0,2886 | 0,2466 | 0,5047 | 0,0033 | 0,2969 | 0,673 |
| **SLAMF1** | 28 | 4,2585 | 0,4706 | 121 | 4,2984 | 0,6679 | 0,7126 | 0,0627 | 0,5247 | 0,0029 | 0,2801 | 0,6896 |
| **CXCL5** | 28 | 9,7638 | 1,1024 | 121 | 9,8798 | 1,41 | 0,6375 | 0,0854 | 0,5508 | 0,0026 | 0,259 | 0,7137 |
| **IL20RA** | 28 | 1,9998 | 0,4592 | 120 | 2,0458 | 0,4862 | 0,6389 | 0,0957 | 0,5764 | 0,0023 | 0,2393 | 0,7365 |
| **CXCL11** | 28 | 8,9644 | 0,7951 | 121 | 9,0288 | 0,9329 | 0,7107 | 0,0708 | 0,6061 | 0,0019 | 0,2174 | 0,7534 |
| **IL5** | 28 | 1,8958 | 0,7047 | 121 | 2,0232 | 1,1093 | 0,4485 | 0,1217 | 0,6104 | 0,0019 | 0,2144 | 0,7534 |
| **IL10** | 28 | 4,7825 | 0,6924 | 121 | 4,6888 | 0,8726 | 0,5432 | 0,1112 | 0,6141 | 0,0018 | 0,2117 | 0,7534 |
| **MCP4** | 28 | 14,1296 | 0,8111 | 121 | 13,9958 | 0,7161 | 0,4268 | 0,1822 | 0,6325 | 0,0017 | 0,1989 | 0,7582 |
| **TNFRSF9** | 28 | 7,7082 | 0,7766 | 121 | 7,5381 | 0,6955 | 0,2938 | 0,2393 | 0,6346 | 0,0016 | 0,1975 | 0,7582 |
| **ST1A1** | 28 | 3,2449 | 0,7524 | 121 | 3,1822 | 0,7286 | 0,6916 | 0,0855 | 0,6592 | 0,0014 | 0,181 | 0,7775 |
| **CCL11** | 28 | 8,9992 | 0,6878 | 121 | 8,9508 | 0,6247 | 0,7348 | 0,076 | 0,7171 | 0,001 | 0,1444 | 0,8271 |
| **CST5** | 28 | 7,0365 | 0,6955 | 121 | 6,9686 | 0,8119 | 0,6544 | 0,0858 | 0,7234 | 0,0009 | 0,1406 | 0,8271 |
| **TNF** | 28 | 3,8792 | 0,5023 | 121 | 3,827 | 0,663 | 0,6441 | 0,0821 | 0,7325 | 0,0008 | 0,1352 | 0,8271 |
| **IL2** | 28 | 1,4957 | 0,2569 | 121 | 1,4697 | 0,2211 | 0,624 | 0,1139 | 0,7372 | 0,0008 | 0,1324 | 0,8271 |
| **FGF5** | 28 | 2,0566 | 0,239 | 121 | 2,0599 | 0,5741 | 0,9628 | 0,0061 | 0,7512 | 0,0007 | 0,1242 | 0,8327 |
| **CD244** | 28 | 7,0908 | 0,4918 | 121 | 7,0502 | 0,5065 | 0,6978 | 0,0805 | 0,769 | 0,0006 | 0,1141 | 0,8422 |
| **CCL19** | 28 | 11,3845 | 0,948 | 121 | 11,3057 | 1,0578 | 0,7002 | 0,0759 | 0,7796 | 0,0006 | 0,1081 | 0,8438 |
| **IL13** | 28 | 1,6189 | 0,5334 | 121 | 1,6349 | 0,895 | 0,9021 | 0,019 | 0,7929 | 0,0005 | 0,1008 | 0,8482 |
| **IL7** | 28 | 2,3881 | 0,6393 | 121 | 2,3614 | 0,57 | 0,8398 | 0,0459 | 0,8396 | 0,0003 | 0,0759 | 0,8878 |
| **SIRT2** | 28 | 5,4949 | 0,8984 | 121 | 5,6058 | 1,2302 | 0,5876 | 0,0943 | 0,853 | 0,0002 | 0,069 | 0,8918 |
| **IL20** | 28 | 1,4055 | 0,238 | 121 | 1,4017 | 0,4368 | 0,9494 | 0,0094 | 0,9178 | 0,0001 | 0,0373 | 0,9487 |
| **MCP2** | 28 | 8,7804 | 0,4115 | 121 | 8,7006 | 0,7256 | 0,4364 | 0,1176 | 0,9333 | 0,0001 | 0,03 | 0,954 |
| **STAMBP** | 28 | 6,0164 | 0,7681 | 121 | 6,0601 | 0,9655 | 0,7976 | 0,0469 | 0,9502 | 0 | 0,0222 | 0,9606 |
| **TSLP** | 28 | 2,304 | 0,5077 | 121 | 2,2893 | 1,0337 | 0,9133 | 0,0153 | 0,9891 | 0 | 0,0047 | 0,9891 |

**Supplementary Table 3) Markers of inflammation correlated to liver-related admissions within 180 days. ANCOVA analysis. 92 markers.** Controlled for sex, age, cancer, depression, psychiatric disorder, diabetes, heart disease, hypertension and hypercholesterolaemia.

| **event** | **Liver-related admission** | | | **No liver-related admission** | | | **T-test** | | **Ancova** | | | |
| --- | --- | --- | --- | --- | --- | --- | --- | --- | --- | --- | --- | --- |
| **stats** | **count** | **mean** | **std** | **count** | **mean** | **std** | **p-val** | **cohen-d** | **p-unc** | **np2** | **-Log10 pvalue** | **qvalue** |
| **variable** |  |  |  |  |  |  |  |  |  |  |  |  |
| **CXCL6** | 45 | 10,2835 | 0,8462 | 97 | 9,8368 | 0,9085 | 0,0053 | 0,5022 | 0,0015 | 0,0747 | 2,836 | 0,1122 |
| **CCL28** | 45 | 4,4261 | 0,6038 | 97 | 4,1831 | 0,5532 | 0,0246 | 0,4267 | 0,0048 | 0,0591 | 2,3173 | 0,1122 |
| **HGF** | 45 | 11,0871 | 0,8723 | 97 | 10,6431 | 0,8654 | 0,0058 | 0,5118 | 0,0056 | 0,0571 | 2,2512 | 0,1122 |
| **CX3CL1** | 45 | 5,9905 | 0,5127 | 97 | 5,7287 | 0,5952 | 0,0085 | 0,4588 | 0,0057 | 0,057 | 2,2474 | 0,1122 |
| **uPA** | 45 | 11,4049 | 0,4918 | 97 | 11,1614 | 0,5205 | 0,0084 | 0,476 | 0,0061 | 0,056 | 2,2148 | 0,1122 |
| **IL8** | 45 | 9,083 | 1,4494 | 97 | 8,4528 | 1,4361 | 0,0178 | 0,4375 | 0,0076 | 0,0532 | 2,1201 | 0,1163 |
| **TRANCE** | 45 | 4,9364 | 0,9164 | 97 | 4,6324 | 0,6684 | 0,0504 | 0,4025 | 0,0102 | 0,0493 | 1,9912 | 0,1341 |
| **TWEAK** | 45 | 8,7993 | 0,5728 | 97 | 8,586 | 0,4792 | 0,0332 | 0,4179 | 0,0128 | 0,0464 | 1,893 | 0,1471 |
| **CCL11** | 45 | 9,1263 | 0,6464 | 97 | 8,8924 | 0,5985 | 0,0433 | 0,381 | 0,0147 | 0,0446 | 1,8321 | 0,1505 |
| **IL15RA** | 45 | 2,785 | 0,6294 | 97 | 2,5946 | 0,4366 | 0,0712 | 0,3768 | 0,0171 | 0,0426 | 1,7664 | 0,1575 |
| **SCF** | 45 | 7,2595 | 0,7892 | 97 | 7,6331 | 0,9514 | 0,0158 | 0,4135 | 0,0197 | 0,0408 | 1,7045 | 0,1651 |
| **CASP8** | 45 | 3,9344 | 0,4875 | 97 | 3,748 | 0,5108 | 0,0396 | 0,3702 | 0,0219 | 0,0394 | 1,6587 | 0,1682 |
| **IL10RB** | 45 | 7,2174 | 0,3051 | 97 | 7,0752 | 0,316 | 0,0124 | 0,4548 | 0,0271 | 0,0367 | 1,5672 | 0,1917 |
| **LIF** | 45 | 1,5036 | 0,8619 | 97 | 1,246 | 0,5297 | 0,0694 | 0,3947 | 0,0314 | 0,0349 | 1,5035 | 0,2061 |
| **IL12B** | 45 | 6,6112 | 1,1985 | 97 | 6,9763 | 1,0078 | 0,0803 | 0,3408 | 0,0364 | 0,033 | 1,4391 | 0,2231 |
| **CSF1** | 45 | 10,9552 | 0,2179 | 97 | 10,8892 | 0,2149 | 0,0956 | 0,3056 | 0,0415 | 0,0314 | 1,3824 | 0,2384 |
| **VEGFA** | 45 | 11,6308 | 0,5174 | 97 | 11,4811 | 0,4945 | 0,1077 | 0,2983 | 0,0514 | 0,0287 | 1,2887 | 0,2784 |
| **CCL4** | 45 | 6,4156 | 0,4231 | 97 | 6,6851 | 1,001 | 0,0258 | 0,3126 | 0,0592 | 0,0269 | 1,2273 | 0,2965 |
| **IL6** | 45 | 5,2495 | 1,1815 | 97 | 4,8547 | 1,243 | 0,0718 | 0,3225 | 0,0612 | 0,0265 | 1,213 | 0,2965 |
| **FGF19** | 45 | 9,9351 | 1,461 | 97 | 9,3585 | 1,3551 | 0,028 | 0,415 | 0,0728 | 0,0244 | 1,1379 | 0,3166 |
| **LAP_TGFb1** | 45 | 7,4217 | 0,4043 | 97 | 7,2993 | 0,4539 | 0,11 | 0,2788 | 0,0754 | 0,0239 | 1,1227 | 0,3166 |
| **IL33** | 45 | 1,3828 | 0,305 | 97 | 1,2843 | 0,2798 | 0,0699 | 0,3421 | 0,0757 | 0,0239 | 1,1209 | 0,3166 |
| **PDL1** | 45 | 6,8695 | 0,4369 | 97 | 6,7316 | 0,4812 | 0,0937 | 0,2948 | 0,0951 | 0,0211 | 1,0216 | 0,3806 |
| **IL18R1** | 45 | 9,8443 | 0,6974 | 97 | 9,6343 | 0,619 | 0,088 | 0,3256 | 0,1088 | 0,0195 | 0,9635 | 0,4112 |
| **CCL20** | 45 | 11,1581 | 1,4245 | 97 | 10,8238 | 1,3568 | 0,1902 | 0,2425 | 0,1117 | 0,0192 | 0,9518 | 0,4112 |
| **IL17A** | 44 | 3,2999 | 0,8151 | 97 | 3,0681 | 1,0116 | 0,151 | 0,2426 | 0,1217 | 0,0183 | 0,9147 | 0,4306 |
| **MCP1** | 45 | 12,283 | 0,5872 | 97 | 12,1748 | 0,565 | 0,3044 | 0,1891 | 0,147 | 0,016 | 0,8327 | 0,4613 |
| **TGFa** | 45 | 3,9985 | 0,5016 | 97 | 3,8695 | 0,5593 | 0,1727 | 0,2381 | 0,1474 | 0,016 | 0,8315 | 0,4613 |
| **CD5** | 45 | 6,4149 | 0,622 | 97 | 6,2957 | 0,5142 | 0,2662 | 0,2167 | 0,1531 | 0,0155 | 0,8151 | 0,4613 |
| **TRAIL** | 45 | 7,2566 | 0,4198 | 97 | 7,3511 | 0,3959 | 0,2072 | 0,2343 | 0,1595 | 0,015 | 0,7972 | 0,4613 |
| **IL18** | 45 | 10,1819 | 0,6662 | 97 | 9,988 | 0,8066 | 0,135 | 0,2534 | 0,1629 | 0,0148 | 0,7881 | 0,4613 |
| **CD8A** | 45 | 11,1816 | 0,7148 | 97 | 10,987 | 0,8612 | 0,1612 | 0,2378 | 0,1632 | 0,0148 | 0,7872 | 0,4613 |
| **CXCL1** | 45 | 9,9265 | 1,0805 | 97 | 9,7017 | 1,1519 | 0,2618 | 0,1989 | 0,1693 | 0,0144 | 0,7713 | 0,4613 |
| **ARTN** | 45 | 2,2462 | 0,5551 | 97 | 2,1126 | 0,7305 | 0,2317 | 0,1964 | 0,1705 | 0,0143 | 0,7683 | 0,4613 |
| **IL13** | 45 | 1,7446 | 0,9664 | 97 | 1,5674 | 0,7878 | 0,2857 | 0,209 | 0,1759 | 0,0139 | 0,7547 | 0,4625 |
| **OSM** | 45 | 4,842 | 1,0186 | 97 | 4,6487 | 0,9658 | 0,2879 | 0,1968 | 0,2062 | 0,0122 | 0,6856 | 0,527 |
| **LIFR** | 45 | 5,1475 | 0,3302 | 97 | 5,052 | 0,364 | 0,1239 | 0,2701 | 0,219 | 0,0115 | 0,6595 | 0,5446 |
| **IL22_RA1** | 45 | 3,0552 | 0,5251 | 97 | 2,9406 | 0,5399 | 0,2334 | 0,2142 | 0,235 | 0,0107 | 0,6288 | 0,5551 |
| **CD6** | 45 | 7,2415 | 0,833 | 97 | 7,0977 | 0,7053 | 0,3188 | 0,1924 | 0,2358 | 0,0107 | 0,6274 | 0,5551 |
| **IL2** | 45 | 1,4294 | 0,1765 | 97 | 1,4893 | 0,2436 | 0,0995 | 0,2669 | 0,2413 | 0,0105 | 0,6174 | 0,5551 |
| **IL20** | 45 | 1,4357 | 0,5544 | 97 | 1,3755 | 0,3251 | 0,5014 | 0,1464 | 0,2628 | 0,0096 | 0,5804 | 0,5811 |
| **CCL23** | 45 | 10,5175 | 0,5452 | 97 | 10,3558 | 0,6602 | 0,1278 | 0,2582 | 0,2653 | 0,0095 | 0,5763 | 0,5811 |
| **OPG** | 45 | 11,3354 | 0,583 | 97 | 11,2613 | 0,3978 | 0,4428 | 0,1595 | 0,2971 | 0,0083 | 0,527 | 0,6177 |
| **4EBP1** | 45 | 9,6349 | 0,9348 | 97 | 9,4772 | 0,9822 | 0,3598 | 0,163 | 0,2978 | 0,0083 | 0,5261 | 0,6177 |
| **CD244** | 45 | 7,1345 | 0,5045 | 97 | 7,0273 | 0,4964 | 0,2398 | 0,2148 | 0,3201 | 0,0075 | 0,4947 | 0,6177 |
| **FGF21** | 45 | 8,2381 | 2,8344 | 97 | 8,0319 | 2,3582 | 0,6723 | 0,0819 | 0,3215 | 0,0075 | 0,4928 | 0,6177 |
| **MCP3** | 45 | 2,5981 | 0,7228 | 97 | 2,4583 | 0,6434 | 0,2706 | 0,2089 | 0,3238 | 0,0074 | 0,4897 | 0,6177 |
| **IL17C** | 45 | 4,2909 | 1,0478 | 97 | 4,1486 | 1,1427 | 0,4664 | 0,1278 | 0,3288 | 0,0073 | 0,4831 | 0,6177 |
| **IL4** | 45 | 1,0836 | 0,583 | 97 | 1,2422 | 0,9745 | 0,2307 | 0,1821 | 0,3401 | 0,0069 | 0,4684 | 0,6177 |
| **CXCL5** | 45 | 10,0159 | 1,2844 | 97 | 9,8389 | 1,4044 | 0,4604 | 0,1294 | 0,3409 | 0,0069 | 0,4674 | 0,6177 |
| **SLAMF1** | 45 | 4,3798 | 0,5705 | 97 | 4,2538 | 0,6677 | 0,2494 | 0,1972 | 0,3424 | 0,0069 | 0,4654 | 0,6177 |
| **BetaNGF** | 45 | 0,3456 | 0,2584 | 97 | 0,3302 | 0,2081 | 0,7269 | 0,0684 | 0,3849 | 0,0058 | 0,4147 | 0,6645 |
| **MCP2** | 45 | 8,6482 | 0,5868 | 97 | 8,7382 | 0,7296 | 0,4343 | 0,1308 | 0,3923 | 0,0056 | 0,4064 | 0,6645 |
| **CST5** | 45 | 7,0176 | 0,8525 | 97 | 6,9173 | 0,7581 | 0,5014 | 0,1272 | 0,4 | 0,0054 | 0,3979 | 0,6645 |
| **IL7** | 45 | 2,298 | 0,4921 | 97 | 2,4005 | 0,6253 | 0,2934 | 0,1746 | 0,4057 | 0,0053 | 0,3918 | 0,6645 |
| **MMP1** | 45 | 14,2985 | 1,2138 | 97 | 14,1874 | 1,0658 | 0,5998 | 0,0997 | 0,41 | 0,0052 | 0,3873 | 0,6645 |
| **FGF23** | 45 | 4,6957 | 1,7776 | 97 | 4,4019 | 1,5759 | 0,3455 | 0,1789 | 0,4117 | 0,0052 | 0,3854 | 0,6645 |
| **IL10RA** | 45 | 2,075 | 0,6105 | 97 | 1,9758 | 0,5697 | 0,3602 | 0,1702 | 0,4587 | 0,0042 | 0,3385 | 0,7275 |
| **CCL19** | 45 | 11,3503 | 1,1182 | 97 | 11,3165 | 1,0264 | 0,8639 | 0,032 | 0,4692 | 0,004 | 0,3286 | 0,7317 |
| **TNFSF14** | 45 | 5,5789 | 0,677 | 97 | 5,4515 | 0,8528 | 0,3402 | 0,1589 | 0,4778 | 0,0039 | 0,3207 | 0,7326 |
| **ST1A1** | 45 | 3,2583 | 0,7135 | 97 | 3,1657 | 0,7601 | 0,4826 | 0,1242 | 0,4897 | 0,0037 | 0,3101 | 0,7374 |
| **IL20RA** | 44 | 2,016 | 0,5027 | 97 | 2,0542 | 0,4844 | 0,6738 | 0,0779 | 0,4973 | 0,0036 | 0,3034 | 0,7374 |
| **CCL25** | 45 | 7,2973 | 0,6786 | 97 | 7,2824 | 0,5752 | 0,899 | 0,0244 | 0,5126 | 0,0033 | 0,2903 | 0,7374 |
| **ADA** | 45 | 6,2626 | 0,6357 | 97 | 6,161 | 0,5486 | 0,3583 | 0,176 | 0,5142 | 0,0033 | 0,2889 | 0,7374 |
| **IL1_a** | 45 | -0,7284 | 0,3731 | 97 | -0,7382 | 0,3922 | 0,8864 | 0,0254 | 0,521 | 0,0032 | 0,2831 | 0,7374 |
| **TNF** | 45 | 3,8762 | 0,6829 | 97 | 3,8211 | 0,6277 | 0,6474 | 0,0854 | 0,5578 | 0,0026 | 0,2535 | 0,7775 |
| **CDCP1** | 45 | 5,1955 | 0,6541 | 97 | 5,1004 | 0,9269 | 0,4842 | 0,1118 | 0,573 | 0,0024 | 0,2418 | 0,7869 |
| **Flt3L** | 45 | 9,5352 | 0,5109 | 97 | 9,6465 | 0,6086 | 0,2592 | 0,192 | 0,5994 | 0,0021 | 0,2223 | 0,7964 |
| **TNFB** | 45 | 5,2211 | 0,656 | 97 | 5,3104 | 0,6613 | 0,4537 | 0,1353 | 0,6025 | 0,0021 | 0,2201 | 0,7964 |
| **MCP4** | 45 | 14,0224 | 0,7686 | 97 | 13,9873 | 0,6695 | 0,7931 | 0,0499 | 0,6059 | 0,002 | 0,2176 | 0,7964 |
| **CXCL9** | 44 | 7,9386 | 0,8383 | 97 | 7,9666 | 1,0881 | 0,8678 | 0,0275 | 0,6264 | 0,0018 | 0,2031 | 0,8043 |
| **GDNF** | 45 | 3,8162 | 0,6323 | 97 | 3,7461 | 0,6267 | 0,539 | 0,1116 | 0,6294 | 0,0018 | 0,201 | 0,8043 |
| **FGF5** | 45 | 2,0741 | 0,4405 | 97 | 2,054 | 0,5791 | 0,8199 | 0,0373 | 0,6794 | 0,0013 | 0,1679 | 0,835 |
| **SIRT2** | 45 | 5,6252 | 1,2704 | 97 | 5,5929 | 1,1632 | 0,8854 | 0,0269 | 0,6819 | 0,0013 | 0,1663 | 0,835 |
| **MMP10** | 45 | 10,048 | 0,7976 | 97 | 10,0868 | 0,7581 | 0,785 | 0,0503 | 0,6881 | 0,0012 | 0,1623 | 0,835 |
| **DNER** | 45 | 8,7809 | 0,408 | 97 | 8,7551 | 0,3713 | 0,7189 | 0,0674 | 0,6898 | 0,0012 | 0,1613 | 0,835 |
| **CCL3** | 45 | 6,9007 | 0,5281 | 97 | 6,934 | 0,8435 | 0,775 | 0,0439 | 0,718 | 0,001 | 0,1439 | 0,8579 |
| **TNFRSF9** | 45 | 7,5646 | 0,7353 | 97 | 7,5297 | 0,6868 | 0,7891 | 0,0496 | 0,7682 | 0,0007 | 0,1145 | 0,8917 |
| **STAMBP** | 45 | 6,0799 | 1,0048 | 97 | 6,0532 | 0,9224 | 0,8799 | 0,0282 | 0,7699 | 0,0007 | 0,1135 | 0,8917 |
| **CD40** | 45 | 12,4214 | 0,503 | 97 | 12,3728 | 0,5332 | 0,6 | 0,0929 | 0,7841 | 0,0006 | 0,1056 | 0,8917 |
| **CXCL10** | 45 | 11,0593 | 0,8702 | 97 | 11,103 | 1,0185 | 0,7927 | 0,0449 | 0,7857 | 0,0006 | 0,1048 | 0,8917 |
| **TSLP** | 45 | 2,2927 | 0,5691 | 97 | 2,3026 | 1,1186 | 0,9446 | 0,0101 | 0,8012 | 0,0005 | 0,0962 | 0,8917 |
| **IL5** | 45 | 1,9936 | 1,1042 | 97 | 1,9993 | 1,0484 | 0,977 | 0,0053 | 0,8045 | 0,0005 | 0,0945 | 0,8917 |
| **ENRAGE** | 45 | 3,5302 | 1,1862 | 97 | 3,4098 | 0,9656 | 0,5533 | 0,1158 | 0,8279 | 0,0004 | 0,082 | 0,9068 |
| **IL10** | 45 | 4,6931 | 0,6254 | 97 | 4,6692 | 0,9143 | 0,8559 | 0,0287 | 0,8574 | 0,0002 | 0,0668 | 0,9214 |
| **IFNg** | 45 | 8,2091 | 1,1713 | 97 | 8,1968 | 1,604 | 0,9591 | 0,0083 | 0,8613 | 0,0002 | 0,0648 | 0,9214 |
| **NRTN** | 45 | 1,4979 | 0,5721 | 97 | 1,4253 | 0,6103 | 0,4929 | 0,1212 | 0,9237 | 0,0001 | 0,0345 | 0,9768 |
| **NT3** | 45 | 2,9823 | 0,68 | 97 | 2,9671 | 0,5146 | 0,8943 | 0,0266 | 0,9393 | 0 | 0,0272 | 0,982 |
| **IL24** | 44 | 2,2459 | 0,5657 | 94 | 2,2799 | 0,7527 | 0,7689 | 0,0486 | 0,9612 | 0 | 0,0172 | 0,9865 |
| **AXIN1** | 45 | 5,4093 | 1,2077 | 97 | 5,3941 | 1,1743 | 0,944 | 0,0128 | 0,965 | 0 | 0,0155 | 0,9865 |
| **IL2RB** | 45 | 1,6957 | 0,817 | 97 | 1,6575 | 0,5646 | 0,7771 | 0,0585 | 0,9914 | 0 | 0,0037 | 0,9916 |
| **CXCL11** | 45 | 9,004 | 0,873 | 97 | 9,0438 | 0,9416 | 0,8061 | 0,0432 | 0,9916 | 0 | 0,0037 | 0,9916 |

**Supplementary Table 4a) Comparisons of the number and causes of deaths in the study group and the validation cohort. b) Comparisons of the number and causes of liver-related admissions in the study group and validation cohort.**

| **Table 4a)** | **Study group (n=149)** | **Validation cohort**  **(n=86)** |
| --- | --- | --- |
| **No. of deaths** | **<181 days** | **<181 days** |
| **Total** | 28 (18.8%) | 9 (10.5%) |
| **Causes:** | | |
| **Liver-related** | 19 | 6 |
| **Non-liver related** | 3 | 0 |
| **Unknown** | 6 | 3 |
| **Scores:** | | |
| **MELD of deceased, n=30, median [range]** | 17 [10;34 | 25 [15;39] |
| **ChildPugh of deceased, n=30, numbers pr group**  **a)**  **b)**  **c)** | 0  13  15 | 0  1  8 |
| **Table 4b)** | **Study group, n=149, 7 excluded, n=142** | **Validation cohort, n=86, 4 excluded, n = 82** |
| **Total admissions** | 126 | 76 |
| **Liver-related** | 84 | 61 |
| **Non-liver related** | 42 | 15 |
| **No. of patients** | 63 (44.3%) (45 liver-related 31.7%) | 32 (39.0%) (26 liver-related 31.7%) |
| **Range of admission pr. Patient** | 1-7 | 1-6 |

**Supplementary Table 5) Markers of inflammation correlated to all-cause death within 180 days in validation cohort.** ANCOVA analysis. 92 markers. Controlled for sex, age, diabetes, heart disease, hypertension and hypercholesterolaemia.

| **event** | **Death in 180 days** | | | **Survival in 180 days** | | | **t-test** | | | **Ancova** | | | |
| --- | --- | --- | --- | --- | --- | --- | --- | --- | --- | --- | --- | --- | --- |
| **stats** | **count** | **mean** | **std** | **count** | **mean** | **std** | **alternative** | **p-val** | **cohen-d** | **p-unc** | **np2** | **-Log10  pvalue** | **qvalue** |
| **variable** |  |  |  |  |  |  |  |  |  |  |  |  |  |
| **IL15RA** | 9 | 3,38 | 0,72 | 77 | 2,40 | 0,53 | two-sided | 0,00 | 1,79 | 0,00 | 0,17 | 3,66 | 0,02 |
| **IL17C** | 9 | 5,35 | 1,57 | 77 | 3,65 | 1,14 | two-sided | 0,01 | 1,43 | 0,00 | 0,15 | 3,19 | 0,03 |
| **IL6** | 9 | 6,60 | 1,88 | 77 | 4,38 | 1,56 | two-sided | 0,01 | 1,40 | 0,00 | 0,12 | 2,61 | 0,08 |
| **CX3CL1** | 9 | 6,49 | 0,74 | 77 | 5,39 | 0,88 | two-sided | 0,00 | 1,28 | 0,01 | 0,09 | 2,08 | 0,19 |
| **TNFSF14** | 9 | 5,82 | 0,50 | 77 | 4,96 | 0,97 | two-sided | 0,00 | 0,92 | 0,01 | 0,08 | 1,88 | 0,22 |
| **TGFa** | 9 | 4,48 | 0,95 | 77 | 3,55 | 0,88 | two-sided | 0,02 | 1,04 | 0,01 | 0,08 | 1,84 | 0,22 |
| **NT3** | 9 | 2,68 | 0,85 | 77 | 2,94 | 0,62 | two-sided | 0,39 | 0,41 | 0,02 | 0,07 | 1,67 | 0,28 |
| **CCL20** | 9 | 11,43 | 1,33 | 77 | 9,97 | 1,87 | two-sided | 0,01 | 0,80 | 0,03 | 0,06 | 1,57 | 0,31 |
| **NRTN** | 9 | 1,03 | 0,22 | 77 | 1,58 | 0,75 | two-sided | 0,00 | 0,76 | 0,04 | 0,06 | 1,45 | 0,36 |
| **IL8** | 9 | 9,07 | 1,18 | 77 | 7,83 | 1,75 | two-sided | 0,01 | 0,73 | 0,05 | 0,05 | 1,32 | 0,44 |
| **IL12B** | 9 | 6,08 | 1,10 | 77 | 6,79 | 1,06 | two-sided | 0,10 | 0,67 | 0,05 | 0,05 | 1,27 | 0,45 |
| **PDL1** | 9 | 7,15 | 0,48 | 77 | 6,41 | 0,91 | two-sided | 0,00 | 0,84 | 0,07 | 0,04 | 1,18 | 0,48 |
| **LIF** | 9 | 1,48 | 1,04 | 77 | 0,87 | 0,49 | two-sided | 0,12 | 1,07 | 0,07 | 0,04 | 1,17 | 0,48 |
| **CCL3** | 9 | 7,22 | 0,48 | 77 | 6,48 | 1,02 | two-sided | 0,00 | 0,75 | 0,08 | 0,04 | 1,10 | 0,50 |
| **CCL4** | 9 | 6,93 | 0,66 | 77 | 6,25 | 0,96 | two-sided | 0,02 | 0,73 | 0,08 | 0,04 | 1,08 | 0,50 |
| **FGF23** | 9 | 5,88 | 1,53 | 77 | 4,42 | 1,93 | two-sided | 0,02 | 0,77 | 0,09 | 0,04 | 1,06 | 0,50 |
| **ENRAGE** | 9 | 3,46 | 1,22 | 77 | 2,82 | 1,05 | two-sided | 0,16 | 0,61 | 0,09 | 0,04 | 1,03 | 0,50 |
| **FGF19** | 9 | 10,35 | 1,44 | 77 | 9,12 | 1,51 | two-sided | 0,04 | 0,82 | 0,11 | 0,03 | 0,94 | 0,58 |
| **IL10RA** | 9 | 2,42 | 1,34 | 77 | 1,78 | 0,82 | two-sided | 0,20 | 0,72 | 0,12 | 0,03 | 0,91 | 0,60 |
| **LIFR** | 9 | 5,45 | 0,67 | 77 | 4,82 | 0,65 | two-sided | 0,02 | 0,97 | 0,14 | 0,03 | 0,86 | 0,60 |
| **IL4** | 9 | 1,22 | 0,65 | 77 | 0,78 | 0,73 | two-sided | 0,08 | 0,61 | 0,14 | 0,03 | 0,86 | 0,60 |
| **CXCL5** | 9 | 9,31 | 1,34 | 77 | 10,57 | 1,85 | two-sided | 0,03 | 0,70 | 0,16 | 0,03 | 0,80 | 0,64 |
| **IL17A** | 9 | 3,10 | 1,10 | 77 | 2,74 | 0,98 | two-sided | 0,38 | 0,35 | 0,18 | 0,02 | 0,75 | 0,64 |
| **OSM** | 9 | 5,16 | 1,02 | 77 | 4,54 | 1,00 | two-sided | 0,12 | 0,61 | 0,18 | 0,02 | 0,74 | 0,64 |
| **IL7** | 9 | 2,00 | 0,30 | 77 | 2,34 | 0,55 | two-sided | 0,01 | 0,63 | 0,19 | 0,02 | 0,72 | 0,64 |
| **CD5** | 9 | 6,54 | 0,71 | 77 | 6,00 | 0,89 | two-sided | 0,06 | 0,61 | 0,19 | 0,02 | 0,72 | 0,64 |
| **VEGFA** | 9 | 11,79 | 0,72 | 77 | 10,98 | 1,39 | two-sided | 0,01 | 0,60 | 0,19 | 0,02 | 0,71 | 0,64 |
| **SCF** | 9 | 7,09 | 0,53 | 77 | 7,52 | 1,24 | two-sided | 0,07 | 0,36 | 0,20 | 0,02 | 0,70 | 0,64 |
| **MMP1** | 9 | 14,82 | 1,17 | 77 | 13,73 | 1,82 | two-sided | 0,03 | 0,62 | 0,20 | 0,02 | 0,69 | 0,64 |
| **MMP10** | 9 | 10,76 | 0,73 | 77 | 9,85 | 1,31 | two-sided | 0,01 | 0,72 | 0,21 | 0,02 | 0,68 | 0,64 |
| **ST1A1** | 9 | 3,91 | 0,74 | 77 | 3,54 | 0,76 | two-sided | 0,19 | 0,48 | 0,22 | 0,02 | 0,66 | 0,66 |
| **CD40** | 9 | 13,18 | 0,80 | 77 | 12,24 | 1,55 | two-sided | 0,01 | 0,63 | 0,23 | 0,02 | 0,63 | 0,67 |
| **MCP4** | 9 | 13,82 | 0,80 | 77 | 14,14 | 1,70 | two-sided | 0,34 | 0,20 | 0,25 | 0,02 | 0,60 | 0,69 |
| **CCL25** | 9 | 7,85 | 0,63 | 77 | 7,07 | 1,05 | two-sided | 0,01 | 0,77 | 0,25 | 0,02 | 0,59 | 0,69 |
| **ARTN** | 9 | 1,90 | 0,76 | 77 | 1,97 | 0,65 | two-sided | 0,80 | 0,10 | 0,27 | 0,02 | 0,57 | 0,70 |
| **TWEAK** | 9 | 8,90 | 1,07 | 77 | 9,12 | 1,45 | two-sided | 0,60 | 0,15 | 0,27 | 0,02 | 0,56 | 0,70 |
| **CST5** | 9 | 7,32 | 0,71 | 77 | 6,68 | 1,08 | two-sided | 0,03 | 0,60 | 0,29 | 0,01 | 0,53 | 0,73 |
| **TNFB** | 9 | 4,98 | 0,80 | 77 | 5,22 | 0,73 | two-sided | 0,42 | 0,32 | 0,33 | 0,01 | 0,49 | 0,76 |
| **CASP8** | 9 | 4,00 | 0,81 | 77 | 3,49 | 0,66 | two-sided | 0,10 | 0,74 | 0,34 | 0,01 | 0,46 | 0,76 |
| **FGF5** | 9 | 2,38 | 0,36 | 77 | 2,04 | 0,57 | two-sided | 0,03 | 0,60 | 0,35 | 0,01 | 0,46 | 0,76 |
| **BetaNGF** | 9 | 0,15 | 0,07 | 77 | 0,19 | 0,14 | two-sided | 0,17 | 0,30 | 0,36 | 0,01 | 0,45 | 0,76 |
| **IL24** | 9 | 2,44 | 0,66 | 77 | 1,98 | 0,68 | two-sided | 0,08 | 0,68 | 0,38 | 0,01 | 0,42 | 0,76 |
| **IL1_a** | 9 | -1,01 | 0,39 | 77 | -0,90 | 0,49 | two-sided | 0,43 | 0,24 | 0,39 | 0,01 | 0,41 | 0,76 |
| **IL5** | 9 | 2,03 | 1,40 | 77 | 1,87 | 1,22 | two-sided | 0,76 | 0,12 | 0,39 | 0,01 | 0,40 | 0,76 |
| **IL10RB** | 9 | 7,09 | 0,28 | 77 | 6,77 | 0,89 | two-sided | 0,03 | 0,38 | 0,41 | 0,01 | 0,39 | 0,76 |
| **TSLP** | 9 | 1,70 | 0,26 | 77 | 1,75 | 0,73 | two-sided | 0,67 | 0,07 | 0,41 | 0,01 | 0,38 | 0,76 |
| **4EBP1** | 9 | 10,23 | 0,95 | 77 | 9,40 | 1,47 | two-sided | 0,04 | 0,58 | 0,42 | 0,01 | 0,37 | 0,76 |
| **Flt3L** | 9 | 9,20 | 0,80 | 77 | 9,29 | 1,03 | two-sided | 0,78 | 0,08 | 0,43 | 0,01 | 0,37 | 0,76 |
| **SLAMF1** | 9 | 4,44 | 0,64 | 77 | 4,12 | 0,86 | two-sided | 0,19 | 0,39 | 0,43 | 0,01 | 0,37 | 0,76 |
| **IFNg** | 9 | 7,24 | 1,16 | 77 | 7,47 | 1,48 | two-sided | 0,60 | 0,16 | 0,43 | 0,01 | 0,36 | 0,76 |
| **TNFRSF9** | 9 | 7,72 | 0,95 | 77 | 7,14 | 1,01 | two-sided | 0,11 | 0,58 | 0,44 | 0,01 | 0,36 | 0,76 |
| **IL18R1** | 9 | 9,56 | 0,62 | 77 | 9,28 | 1,13 | two-sided | 0,27 | 0,26 | 0,44 | 0,01 | 0,35 | 0,76 |
| **MCP3** | 9 | 2,53 | 0,72 | 77 | 2,36 | 0,79 | two-sided | 0,53 | 0,21 | 0,45 | 0,01 | 0,35 | 0,76 |
| **IL20RA** | 9 | 2,13 | 0,41 | 77 | 1,79 | 0,86 | two-sided | 0,06 | 0,41 | 0,45 | 0,01 | 0,34 | 0,76 |
| **SIRT2** | 9 | 5,80 | 0,97 | 77 | 5,91 | 1,47 | two-sided | 0,79 | 0,07 | 0,46 | 0,01 | 0,34 | 0,76 |
| **CCL23** | 9 | 10,53 | 0,77 | 77 | 10,02 | 1,41 | two-sided | 0,11 | 0,37 | 0,48 | 0,01 | 0,32 | 0,76 |
| **LAP_TGFb1** | 9 | 7,23 | 0,36 | 77 | 6,97 | 0,97 | two-sided | 0,13 | 0,28 | 0,48 | 0,01 | 0,32 | 0,76 |
| **MCP1** | 9 | 12,24 | 0,68 | 77 | 11,69 | 1,50 | two-sided | 0,07 | 0,38 | 0,49 | 0,01 | 0,31 | 0,76 |
| **CDCP1** | 9 | 4,68 | 0,63 | 77 | 4,38 | 0,99 | two-sided | 0,23 | 0,31 | 0,49 | 0,01 | 0,31 | 0,76 |
| **CXCL6** | 9 | 10,13 | 0,81 | 77 | 9,75 | 1,38 | two-sided | 0,25 | 0,28 | 0,50 | 0,01 | 0,30 | 0,76 |
| **CSF1** | 9 | 10,84 | 0,19 | 77 | 10,50 | 1,08 | two-sided | 0,02 | 0,33 | 0,51 | 0,01 | 0,29 | 0,76 |
| **IL10** | 9 | 4,63 | 0,68 | 77 | 4,24 | 0,94 | two-sided | 0,15 | 0,42 | 0,51 | 0,01 | 0,29 | 0,76 |
| **IL18** | 9 | 9,89 | 1,13 | 77 | 9,47 | 1,19 | two-sided | 0,32 | 0,35 | 0,56 | 0,00 | 0,25 | 0,82 |
| **CD6** | 9 | 7,16 | 0,73 | 77 | 6,68 | 0,93 | two-sided | 0,10 | 0,52 | 0,59 | 0,00 | 0,23 | 0,84 |
| **uPA** | 9 | 11,24 | 0,32 | 77 | 10,72 | 1,39 | two-sided | 0,01 | 0,39 | 0,59 | 0,00 | 0,23 | 0,84 |
| **CCL28** | 9 | 4,41 | 0,73 | 77 | 4,29 | 0,76 | two-sided | 0,67 | 0,15 | 0,62 | 0,00 | 0,20 | 0,87 |
| **STAMBP** | 9 | 6,33 | 0,70 | 77 | 6,40 | 1,18 | two-sided | 0,82 | 0,06 | 0,65 | 0,00 | 0,19 | 0,87 |
| **TRANCE** | 9 | 4,52 | 1,19 | 77 | 4,48 | 0,94 | two-sided | 0,92 | 0,05 | 0,65 | 0,00 | 0,19 | 0,87 |
| **IL2** | 9 | 1,39 | 0,23 | 77 | 1,46 | 0,33 | two-sided | 0,38 | 0,24 | 0,65 | 0,00 | 0,18 | 0,87 |
| **TRAIL** | 9 | 6,97 | 0,20 | 77 | 7,07 | 1,04 | two-sided | 0,45 | 0,10 | 0,66 | 0,00 | 0,18 | 0,87 |
| **CD244** | 9 | 7,43 | 0,53 | 77 | 7,11 | 0,82 | two-sided | 0,13 | 0,41 | 0,67 | 0,00 | 0,17 | 0,87 |
| **AXIN1** | 9 | 5,24 | 1,08 | 77 | 5,68 | 1,30 | two-sided | 0,29 | 0,34 | 0,70 | 0,00 | 0,16 | 0,88 |
| **MCP2** | 9 | 8,38 | 0,51 | 77 | 8,44 | 1,26 | two-sided | 0,79 | 0,05 | 0,70 | 0,00 | 0,15 | 0,88 |
| **CCL19** | 9 | 11,15 | 1,14 | 77 | 10,78 | 1,83 | two-sided | 0,41 | 0,21 | 0,74 | 0,00 | 0,13 | 0,90 |
| **OPG** | 9 | 11,14 | 0,34 | 77 | 10,91 | 1,35 | two-sided | 0,24 | 0,18 | 0,74 | 0,00 | 0,13 | 0,90 |
| **IL22_RA1** | 9 | 2,80 | 0,36 | 76 | 2,72 | 0,78 | two-sided | 0,56 | 0,12 | 0,74 | 0,00 | 0,13 | 0,90 |
| **IL33** | 9 | 1,77 | 0,51 | 77 | 1,52 | 0,43 | two-sided | 0,19 | 0,57 | 0,77 | 0,00 | 0,11 | 0,93 |
| **FGF21** | 9 | 7,43 | 2,42 | 77 | 7,36 | 2,54 | two-sided | 0,93 | 0,03 | 0,79 | 0,00 | 0,10 | 0,94 |
| **IL13** | 9 | 1,29 | 1,12 | 77 | 1,32 | 0,89 | two-sided | 0,94 | 0,03 | 0,80 | 0,00 | 0,09 | 0,94 |
| **CXCL10** | 9 | 11,09 | 1,28 | 77 | 10,69 | 1,43 | two-sided | 0,39 | 0,29 | 0,82 | 0,00 | 0,09 | 0,94 |
| **ADA** | 9 | 6,55 | 1,06 | 77 | 6,08 | 0,95 | two-sided | 0,24 | 0,49 | 0,83 | 0,00 | 0,08 | 0,94 |
| **CD8A** | 9 | 10,60 | 0,99 | 77 | 10,79 | 1,49 | two-sided | 0,61 | 0,13 | 0,85 | 0,00 | 0,07 | 0,95 |
| **GDNF** | 9 | 3,83 | 0,74 | 77 | 3,71 | 0,80 | two-sided | 0,64 | 0,16 | 0,87 | 0,00 | 0,06 | 0,96 |
| **CCL11** | 9 | 8,85 | 0,75 | 77 | 8,65 | 1,12 | two-sided | 0,50 | 0,18 | 0,89 | 0,00 | 0,05 | 0,96 |
| **DNER** | 9 | 8,87 | 0,36 | 77 | 8,58 | 0,99 | two-sided | 0,09 | 0,31 | 0,89 | 0,00 | 0,05 | 0,96 |
| **CXCL9** | 9 | 8,60 | 1,56 | 77 | 8,03 | 1,34 | two-sided | 0,32 | 0,42 | 0,92 | 0,00 | 0,04 | 0,97 |
| **IL2RB** | 9 | 1,47 | 0,33 | 77 | 1,46 | 0,56 | two-sided | 0,92 | 0,02 | 0,93 | 0,00 | 0,03 | 0,97 |
| **CXCL1** | 9 | 9,58 | 0,52 | 77 | 9,67 | 1,27 | two-sided | 0,70 | 0,07 | 0,93 | 0,00 | 0,03 | 0,97 |
| **TNF** | 9 | 3,48 | 0,54 | 77 | 3,40 | 0,81 | two-sided | 0,73 | 0,09 | 0,95 | 0,00 | 0,02 | 0,98 |
| **HGF** | 9 | 11,37 | 0,70 | 77 | 11,17 | 1,71 | two-sided | 0,52 | 0,12 | 0,97 | 0,00 | 0,01 | 0,98 |
| **IL20** | 9 | 1,35 | 0,32 | 77 | 1,37 | 0,71 | two-sided | 0,88 | 0,03 | 0,97 | 0,00 | 0,01 | 0,98 |
| **CXCL11** | 9 | 8,80 | 0,78 | 77 | 8,67 | 1,40 | two-sided | 0,68 | 0,10 | 0,99 | 0,00 | 0,01 | 0,99 |

**Supplementary Table 6) Markers of inflammation correlated to liver-related admissions within 180 days in the validation cohort**. ANCOVA analysis. 92 markers. Controlled for sex, age, diabetes, heart disease, hypertension and hypercholesterolaemia.

| **event** | **Liver-related admission** | | | **No liver-related admission** | | | **ttest** | | | **ancova** | | | |
| --- | --- | --- | --- | --- | --- | --- | --- | --- | --- | --- | --- | --- | --- |
| **stats** | **count** | **mean** | **std** | **count** | **mean** | **std** | **alternative** | **p-val** | **cohen-d** | **p-unc** | **np2** | **-Log10  pvalue** | **qvalue** |
| **variable** |  |  |  |  |  |  |  |  |  |  |  |  |  |
| **IL6** | 26 | 5,49 | 1,22 | 56 | 3,96 | 1,44 | two-sided | 0,00 | 1,11 | 0,00 | 0,16 | 3,25 | 0,05 |
| **TNFSF14** | 26 | 5,48 | 0,66 | 56 | 4,79 | 1,00 | two-sided | 0,00 | 0,77 | 0,01 | 0,08 | 1,84 | 0,51 |
| **TGFa** | 26 | 3,99 | 0,62 | 56 | 3,37 | 0,89 | two-sided | 0,00 | 0,76 | 0,04 | 0,06 | 1,44 | 0,51 |
| **CX3CL1** | 26 | 5,85 | 0,70 | 56 | 5,25 | 0,91 | two-sided | 0,00 | 0,71 | 0,04 | 0,06 | 1,39 | 0,51 |
| **IL10** | 26 | 4,59 | 0,76 | 56 | 4,09 | 0,95 | two-sided | 0,01 | 0,56 | 0,04 | 0,06 | 1,35 | 0,51 |
| **GDNF** | 26 | 3,94 | 0,66 | 56 | 3,60 | 0,84 | two-sided | 0,05 | 0,43 | 0,06 | 0,05 | 1,26 | 0,51 |
| **CCL3** | 26 | 6,91 | 0,59 | 56 | 6,33 | 1,09 | two-sided | 0,00 | 0,61 | 0,07 | 0,05 | 1,17 | 0,51 |
| **LIFR** | 26 | 5,10 | 0,41 | 56 | 4,74 | 0,71 | two-sided | 0,01 | 0,56 | 0,07 | 0,05 | 1,17 | 0,51 |
| **ARTN** | 26 | 1,75 | 0,52 | 56 | 2,04 | 0,68 | two-sided | 0,04 | 0,45 | 0,07 | 0,04 | 1,14 | 0,51 |
| **PDL1** | 26 | 6,79 | 0,52 | 56 | 6,28 | 0,98 | two-sided | 0,00 | 0,60 | 0,08 | 0,04 | 1,09 | 0,51 |
| **IL13** | 26 | 1,57 | 1,04 | 56 | 1,22 | 0,86 | two-sided | 0,15 | 0,38 | 0,08 | 0,04 | 1,08 | 0,51 |
| **FGF23** | 26 | 5,24 | 1,87 | 56 | 4,14 | 1,87 | two-sided | 0,02 | 0,59 | 0,08 | 0,04 | 1,07 | 0,51 |
| **CCL20** | 26 | 10,65 | 1,34 | 56 | 9,72 | 1,98 | two-sided | 0,02 | 0,51 | 0,09 | 0,04 | 1,07 | 0,51 |
| **IL15RA** | 26 | 2,65 | 0,60 | 56 | 2,33 | 0,49 | two-sided | 0,02 | 0,60 | 0,09 | 0,04 | 1,05 | 0,51 |
| **MMP1** | 26 | 14,39 | 1,00 | 56 | 13,51 | 2,01 | two-sided | 0,01 | 0,50 | 0,09 | 0,04 | 1,03 | 0,51 |
| **CCL4** | 26 | 6,59 | 0,55 | 56 | 6,17 | 1,08 | two-sided | 0,02 | 0,44 | 0,10 | 0,04 | 1,02 | 0,51 |
| **CCL25** | 26 | 7,44 | 0,53 | 56 | 6,97 | 1,17 | two-sided | 0,01 | 0,47 | 0,10 | 0,04 | 1,02 | 0,51 |
| **MMP10** | 26 | 10,34 | 0,77 | 56 | 9,69 | 1,43 | two-sided | 0,01 | 0,52 | 0,10 | 0,04 | 1,00 | 0,51 |
| **IL10RA** | 26 | 2,02 | 1,04 | 56 | 1,74 | 0,82 | two-sided | 0,24 | 0,31 | 0,11 | 0,04 | 0,95 | 0,53 |
| **IL8** | 26 | 8,49 | 1,44 | 56 | 7,60 | 1,79 | two-sided | 0,02 | 0,53 | 0,12 | 0,03 | 0,94 | 0,53 |
| **IL7** | 26 | 2,13 | 0,37 | 56 | 2,40 | 0,60 | two-sided | 0,01 | 0,50 | 0,13 | 0,03 | 0,88 | 0,55 |
| **CCL11** | 26 | 8,91 | 0,63 | 56 | 8,57 | 1,25 | two-sided | 0,10 | 0,31 | 0,13 | 0,03 | 0,88 | 0,55 |
| **CXCL5** | 26 | 9,89 | 1,24 | 56 | 10,82 | 1,99 | two-sided | 0,01 | 0,52 | 0,15 | 0,03 | 0,84 | 0,58 |
| **CST5** | 26 | 7,03 | 0,64 | 56 | 6,56 | 1,19 | two-sided | 0,02 | 0,45 | 0,16 | 0,03 | 0,79 | 0,59 |
| **FGF19** | 26 | 9,57 | 1,10 | 56 | 8,96 | 1,62 | two-sided | 0,05 | 0,41 | 0,18 | 0,03 | 0,76 | 0,59 |
| **IL17A** | 26 | 3,00 | 0,91 | 56 | 2,66 | 1,00 | two-sided | 0,13 | 0,35 | 0,18 | 0,03 | 0,75 | 0,59 |
| **uPA** | 26 | 11,06 | 0,38 | 56 | 10,60 | 1,60 | two-sided | 0,04 | 0,35 | 0,18 | 0,03 | 0,75 | 0,59 |
| **CD244** | 26 | 7,30 | 0,54 | 56 | 7,06 | 0,90 | two-sided | 0,15 | 0,29 | 0,19 | 0,02 | 0,73 | 0,59 |
| **IL18** | 26 | 9,79 | 0,72 | 56 | 9,32 | 1,32 | two-sided | 0,04 | 0,40 | 0,19 | 0,02 | 0,72 | 0,59 |
| **CCL23** | 26 | 10,46 | 0,60 | 56 | 9,82 | 1,57 | two-sided | 0,01 | 0,48 | 0,19 | 0,02 | 0,72 | 0,59 |
| **SIRT2** | 26 | 5,55 | 1,10 | 56 | 6,05 | 1,54 | two-sided | 0,10 | 0,35 | 0,21 | 0,02 | 0,67 | 0,62 |
| **CD8A** | 26 | 11,05 | 0,68 | 56 | 10,65 | 1,69 | two-sided | 0,13 | 0,28 | 0,22 | 0,02 | 0,67 | 0,62 |
| **CXCL6** | 26 | 10,12 | 0,66 | 56 | 9,62 | 1,54 | two-sided | 0,04 | 0,38 | 0,24 | 0,02 | 0,63 | 0,65 |
| **CDCP1** | 26 | 4,57 | 0,77 | 56 | 4,29 | 1,04 | two-sided | 0,18 | 0,29 | 0,24 | 0,02 | 0,62 | 0,65 |
| **BetaNGF** | 26 | 0,16 | 0,06 | 56 | 0,20 | 0,16 | two-sided | 0,09 | 0,31 | 0,25 | 0,02 | 0,60 | 0,66 |
| **STAMBP** | 26 | 6,14 | 0,77 | 56 | 6,51 | 1,27 | two-sided | 0,11 | 0,32 | 0,28 | 0,02 | 0,56 | 0,66 |
| **SCF** | 26 | 7,30 | 0,74 | 56 | 7,56 | 1,39 | two-sided | 0,27 | 0,22 | 0,28 | 0,02 | 0,55 | 0,66 |
| **VEGFA** | 26 | 11,41 | 0,63 | 56 | 10,81 | 1,56 | two-sided | 0,02 | 0,44 | 0,29 | 0,02 | 0,54 | 0,66 |
| **SLAMF1** | 26 | 4,35 | 0,61 | 56 | 4,02 | 0,92 | two-sided | 0,05 | 0,40 | 0,29 | 0,02 | 0,54 | 0,66 |
| **CSF1** | 26 | 10,76 | 0,20 | 56 | 10,40 | 1,25 | two-sided | 0,04 | 0,35 | 0,29 | 0,02 | 0,54 | 0,66 |
| **MCP3** | 26 | 2,58 | 0,75 | 56 | 2,26 | 0,78 | two-sided | 0,08 | 0,41 | 0,30 | 0,02 | 0,52 | 0,67 |
| **TNF** | 26 | 3,58 | 0,60 | 56 | 3,29 | 0,86 | two-sided | 0,08 | 0,37 | 0,31 | 0,01 | 0,51 | 0,67 |
| **ADA** | 26 | 6,19 | 0,58 | 56 | 6,03 | 1,04 | two-sided | 0,39 | 0,17 | 0,32 | 0,01 | 0,49 | 0,67 |
| **CD40** | 26 | 12,65 | 0,66 | 56 | 12,09 | 1,76 | two-sided | 0,04 | 0,37 | 0,34 | 0,01 | 0,47 | 0,67 |
| **IL10RB** | 26 | 6,99 | 0,38 | 56 | 6,68 | 1,00 | two-sided | 0,04 | 0,37 | 0,34 | 0,01 | 0,47 | 0,67 |
| **MCP1** | 26 | 12,05 | 0,67 | 56 | 11,52 | 1,68 | two-sided | 0,05 | 0,37 | 0,34 | 0,01 | 0,47 | 0,67 |
| **TNFRSF9** | 26 | 7,40 | 0,72 | 56 | 7,01 | 1,08 | two-sided | 0,06 | 0,40 | 0,34 | 0,01 | 0,46 | 0,67 |
| **CXCL11** | 26 | 8,92 | 1,14 | 56 | 8,56 | 1,45 | two-sided | 0,23 | 0,26 | 0,36 | 0,01 | 0,45 | 0,67 |
| **CASP8** | 26 | 3,61 | 0,42 | 56 | 3,46 | 0,73 | two-sided | 0,25 | 0,23 | 0,36 | 0,01 | 0,44 | 0,67 |
| **OSM** | 26 | 4,81 | 0,75 | 56 | 4,44 | 1,06 | two-sided | 0,07 | 0,38 | 0,36 | 0,01 | 0,44 | 0,67 |
| **OPG** | 26 | 11,16 | 0,39 | 56 | 10,80 | 1,55 | two-sided | 0,12 | 0,27 | 0,39 | 0,01 | 0,41 | 0,69 |
| **CD6** | 26 | 6,77 | 0,67 | 56 | 6,68 | 1,02 | two-sided | 0,65 | 0,09 | 0,40 | 0,01 | 0,40 | 0,69 |
| **IL18R1** | 26 | 9,48 | 0,53 | 56 | 9,19 | 1,27 | two-sided | 0,14 | 0,27 | 0,41 | 0,01 | 0,39 | 0,69 |
| **IL17C** | 26 | 4,05 | 1,32 | 56 | 3,53 | 1,06 | two-sided | 0,09 | 0,45 | 0,41 | 0,01 | 0,39 | 0,69 |
| **IL22_RA1** | 26 | 2,62 | 0,79 | 55 | 2,77 | 0,75 | two-sided | 0,43 | 0,19 | 0,42 | 0,01 | 0,38 | 0,69 |
| **LAP_TGFb1** | 26 | 7,17 | 0,47 | 56 | 6,89 | 1,09 | two-sided | 0,11 | 0,29 | 0,42 | 0,01 | 0,38 | 0,69 |
| **IL20RA** | 26 | 1,97 | 0,57 | 56 | 1,74 | 0,94 | two-sided | 0,17 | 0,27 | 0,44 | 0,01 | 0,36 | 0,71 |
| **TSLP** | 26 | 1,66 | 0,46 | 56 | 1,80 | 0,79 | two-sided | 0,31 | 0,20 | 0,45 | 0,01 | 0,34 | 0,72 |
| **CXCL9** | 26 | 7,94 | 0,99 | 56 | 8,07 | 1,45 | two-sided | 0,63 | 0,10 | 0,48 | 0,01 | 0,32 | 0,75 |
| **DNER** | 26 | 8,69 | 0,36 | 56 | 8,55 | 1,14 | two-sided | 0,39 | 0,15 | 0,49 | 0,01 | 0,31 | 0,76 |
| **TRANCE** | 26 | 4,50 | 0,79 | 56 | 4,48 | 0,98 | two-sided | 0,94 | 0,02 | 0,51 | 0,01 | 0,29 | 0,77 |
| **TNFB** | 26 | 5,15 | 0,62 | 56 | 5,27 | 0,77 | two-sided | 0,45 | 0,17 | 0,55 | 0,01 | 0,26 | 0,81 |
| **NRTN** | 26 | 1,41 | 0,59 | 56 | 1,59 | 0,81 | two-sided | 0,24 | 0,25 | 0,56 | 0,00 | 0,25 | 0,81 |
| **NT3** | 26 | 2,92 | 0,60 | 56 | 2,94 | 0,67 | two-sided | 0,87 | 0,04 | 0,59 | 0,00 | 0,23 | 0,81 |
| **IL2RB** | 26 | 1,49 | 0,45 | 56 | 1,43 | 0,58 | two-sided | 0,61 | 0,11 | 0,59 | 0,00 | 0,23 | 0,81 |
| **IFNg** | 26 | 7,35 | 1,20 | 56 | 7,49 | 1,58 | two-sided | 0,66 | 0,09 | 0,60 | 0,00 | 0,22 | 0,81 |
| **IL5** | 26 | 1,61 | 0,71 | 56 | 1,96 | 1,35 | two-sided | 0,13 | 0,29 | 0,60 | 0,00 | 0,22 | 0,81 |
| **FGF5** | 26 | 2,15 | 0,41 | 56 | 2,02 | 0,62 | two-sided | 0,25 | 0,24 | 0,62 | 0,00 | 0,21 | 0,81 |
| **LIF** | 26 | 0,96 | 0,45 | 56 | 0,84 | 0,51 | two-sided | 0,27 | 0,25 | 0,62 | 0,00 | 0,21 | 0,81 |
| **ST1A1** | 26 | 3,51 | 0,84 | 56 | 3,58 | 0,72 | two-sided | 0,71 | 0,09 | 0,62 | 0,00 | 0,21 | 0,81 |
| **CCL28** | 26 | 4,31 | 0,57 | 56 | 4,29 | 0,82 | two-sided | 0,86 | 0,04 | 0,63 | 0,00 | 0,20 | 0,81 |
| **IL2** | 26 | 1,41 | 0,27 | 56 | 1,47 | 0,35 | two-sided | 0,42 | 0,17 | 0,64 | 0,00 | 0,20 | 0,81 |
| **CD5** | 26 | 6,16 | 0,56 | 56 | 5,94 | 0,98 | two-sided | 0,19 | 0,26 | 0,65 | 0,00 | 0,19 | 0,81 |
| **AXIN1** | 26 | 5,41 | 1,28 | 56 | 5,79 | 1,27 | two-sided | 0,21 | 0,30 | 0,72 | 0,00 | 0,14 | 0,90 |
| **Flt3L** | 26 | 9,32 | 0,66 | 56 | 9,26 | 1,16 | two-sided | 0,75 | 0,06 | 0,77 | 0,00 | 0,11 | 0,94 |
| **HGF** | 26 | 11,20 | 1,03 | 56 | 11,17 | 1,90 | two-sided | 0,93 | 0,02 | 0,82 | 0,00 | 0,08 | 0,98 |
| **CCL19** | 26 | 10,87 | 0,99 | 56 | 10,71 | 2,05 | two-sided | 0,64 | 0,09 | 0,82 | 0,00 | 0,08 | 0,98 |
| **TRAIL** | 26 | 7,02 | 0,31 | 56 | 7,09 | 1,20 | two-sided | 0,68 | 0,07 | 0,84 | 0,00 | 0,07 | 1,00 |
| **TWEAK** | 26 | 8,97 | 0,83 | 56 | 9,19 | 1,62 | two-sided | 0,42 | 0,15 | 0,87 | 0,00 | 0,06 | 1,00 |
| **MCP2** | 26 | 8,43 | 0,71 | 56 | 8,42 | 1,41 | two-sided | 0,96 | 0,01 | 0,89 | 0,00 | 0,05 | 1,00 |
| **MCP4** | 26 | 14,14 | 0,92 | 56 | 14,11 | 1,91 | two-sided | 0,94 | 0,01 | 0,90 | 0,00 | 0,05 | 1,00 |
| **IL33** | 26 | 1,54 | 0,36 | 56 | 1,53 | 0,46 | two-sided | 0,89 | 0,03 | 0,91 | 0,00 | 0,04 | 1,00 |
| **FGF21** | 26 | 7,66 | 2,53 | 56 | 7,12 | 2,50 | two-sided | 0,37 | 0,21 | 0,91 | 0,00 | 0,04 | 1,00 |
| **IL12B** | 26 | 6,69 | 0,98 | 56 | 6,75 | 1,13 | two-sided | 0,82 | 0,05 | 0,93 | 0,00 | 0,03 | 1,00 |
| **IL24** | 26 | 2,08 | 0,50 | 56 | 1,95 | 0,73 | two-sided | 0,37 | 0,19 | 0,95 | 0,00 | 0,02 | 1,00 |
| **IL20** | 26 | 1,36 | 0,47 | 56 | 1,38 | 0,77 | two-sided | 0,86 | 0,03 | 0,95 | 0,00 | 0,02 | 1,00 |
| **IL1_a** | 26 | -0,91 | 0,48 | 56 | -0,91 | 0,50 | two-sided | 0,99 | 0,00 | 0,96 | 0,00 | 0,02 | 1,00 |
| **IL4** | 26 | 0,83 | 0,81 | 56 | 0,77 | 0,70 | two-sided | 0,77 | 0,07 | 0,97 | 0,00 | 0,01 | 1,00 |
| **CXCL1** | 26 | 9,61 | 0,75 | 56 | 9,69 | 1,41 | two-sided | 0,75 | 0,06 | 0,98 | 0,00 | 0,01 | 1,00 |
| **ENRAGE** | 26 | 2,95 | 1,16 | 56 | 2,79 | 1,04 | two-sided | 0,55 | 0,15 | 0,98 | 0,00 | 0,01 | 1,00 |
| **4EBP1** | 26 | 9,58 | 0,86 | 56 | 9,37 | 1,65 | two-sided | 0,45 | 0,15 | 0,99 | 0,00 | 0,01 | 1,00 |
| **CXCL10** | 26 | 10,74 | 1,14 | 56 | 10,67 | 1,52 | two-sided | 0,81 | 0,05 | 1,00 | 0,00 | 0,00 | 1,00 |

**Supplementary Figures**

**Supplementary Figure 1)** **Cross-validation analysis with 180-day mortality as endpoint**. Linear correlations of variables. Each intersection of a row and a column shows how these two variables correlate using Pearson correlation. Blue colour indicates positive correlation, red colour indicates negative correlation. Color strength and size of the correlation square indicate the grade of correlation. The higher the saturation of the colour, the larger the correlation magnitude.


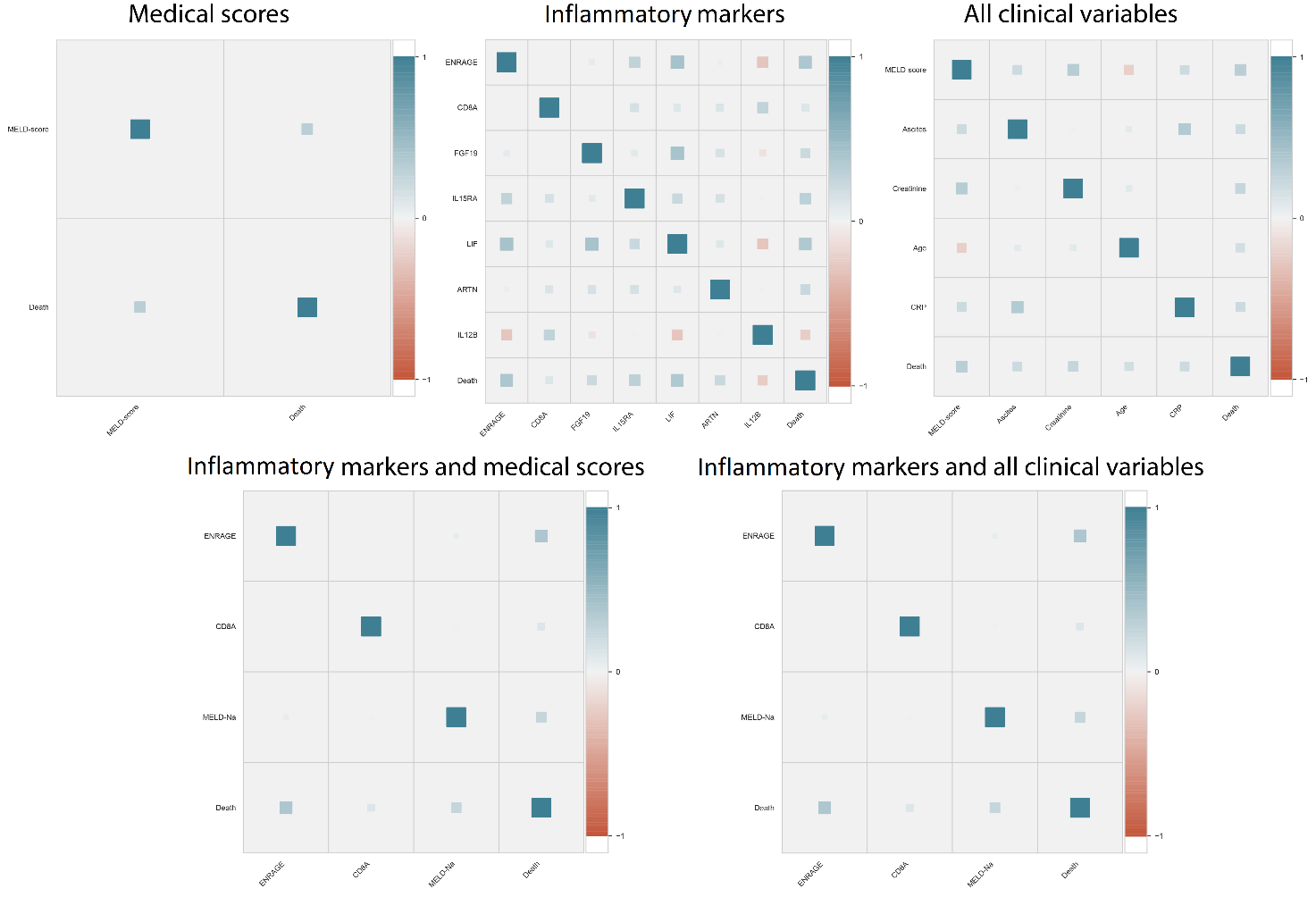


**Supplementary Figure 2) Plot for the cross-validations with 180-day liver-related admission as endpoint.** Linear correlations of variables. Each intersection of a row and a column shows how these two variables correlate using Pearson correlation. Blue colour indicates positive correlation, red colour indicates negative correlation. Colour strength and size of the correlation square indicate the grade of correlation. The stronger the colour, the larger the correlation magnitude.


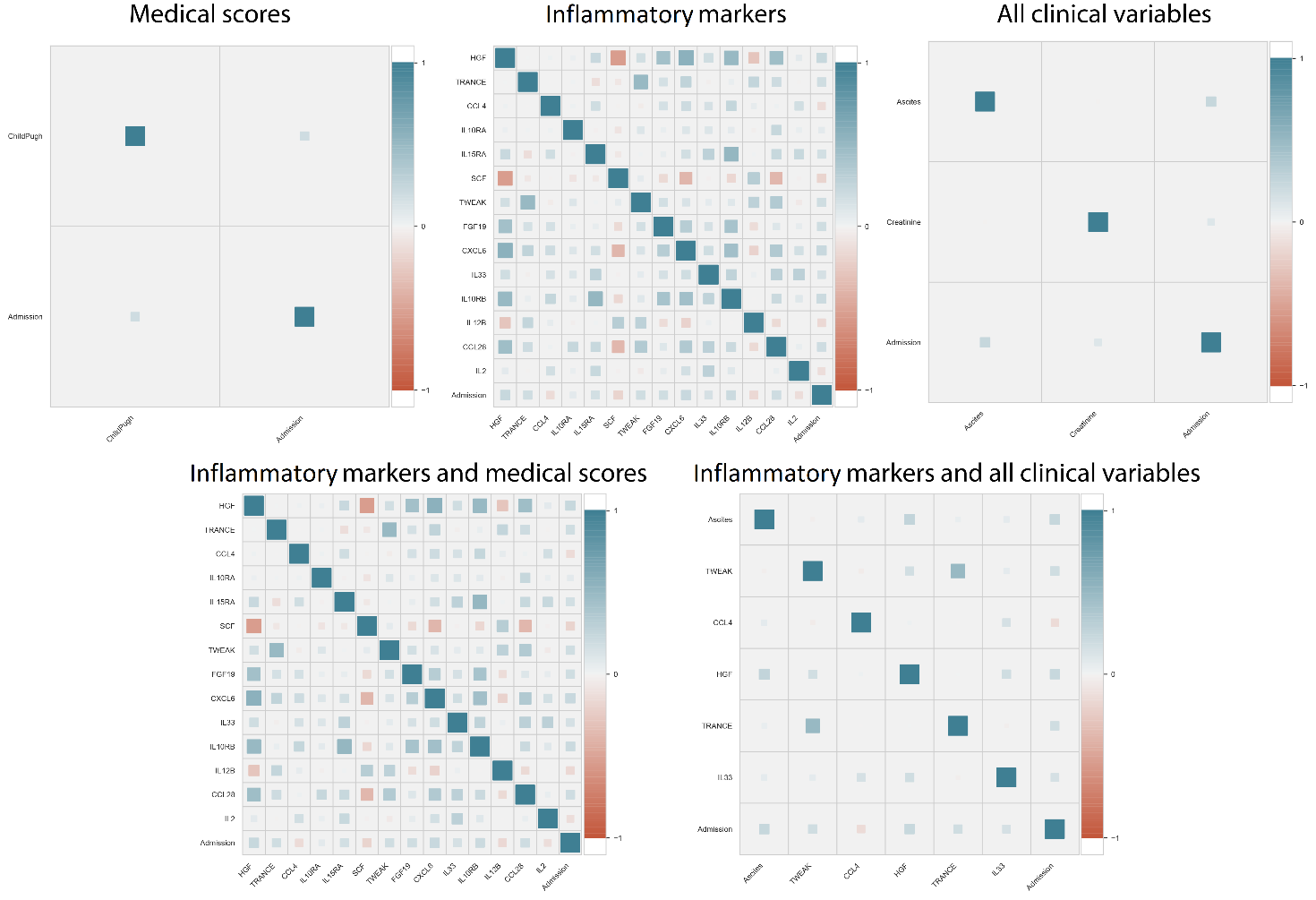


**Supplementary Figure 3) Receiver operating curves of predicting 180-day mortality based on MELD in the study group (blue) and validation cohort (orange).** AUC: area under the curve


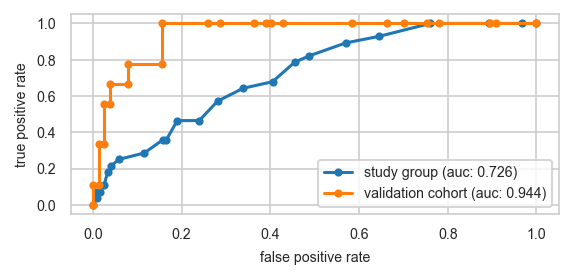

Supplement: Supplementary file 11 — Supplementary Information 11. [file 41598_2023_47384_MOESM11_ESM.docx]
